# Extracellular endonucleases in the midgut of *Myzus persicae* may limit the efficacy of orally delivered RNAi

**DOI:** 10.1038/s41598-019-47357-4

**Published:** 2019-08-15

**Authors:** Amol Bharat Ghodke, Robert Trygve Good, John F. Golz, Derek A. Russell, Owain Edwards, Charles Robin

**Affiliations:** 1 School of BioSciences, The University of Melbourne, Melbourne, Australia; 2 Faculty of Veterinary and Agricultural Sciences, The University of Melbourne, Melbourne, Australia; 3 CSIRO Land and Water, Perth, Australia

**Keywords:** Agricultural genetics, RNAi

## Abstract

*Myzus persicae* is a major pest of many crops including canola and Brassica vegetables, partly because it vectors plant viruses. Previously it has been reported that double-stranded RNA delivered to aphids by injection, artificial diet or transgenic plants has knocked down target genes and caused phenotypic effects. While these studies suggest that RNA interference (RNAi) might be used to suppress aphid populations, none have shown effects sufficient for field control. The current study analyses the efficacy of dsRNA directed against previously reported gene-targets on Green peach aphid (*Myzus persicae*) strains. No silencing effect was observed when dsRNA was delivered in artificial diet with or without transfection reagents. dsRNA produced *in planta* also failed to induce significant RNAi in *M*. *persicae*. Transcriptome analyses of the midgut suggested other potential targets including the Ferritin heavy chain transcripts, but they also could not be knocked down with dsRNA. Here we show that dsRNA is rapidly degraded by midgut secretions of *Myzus persicae*. Analysis of the transcriptome of the *M*. *persicae* midgut revealed that an ortholog of RNases from other insects was abundant.

## Introduction

In 2007, two independent studies reported the successful use of dietary-delivered RNA interference (*RNAi*) to impair pest insect growth^1,2^. These studies held out the prospect of a wealth of new possible transgene-based insecticides that could be used to make numerous crops resistant to specific insect pests^3^. As RNAi works via sequence identity, transgenes can be designed to selectively target pest species without affecting other organisms in the environment^4–6^. One of the studies reported that beetles (*Diabrotica virgifera virgifera*) fed an artificial diet containing double-stranded RNA (dsRNA) had their target genes silenced in a sequence-dependent fashion and their development disrupted^1^. It also showed that transgenic plants expressing dsRNA were less damaged by beetles than control plants. The other study reported that plants producing a particular dsRNA significantly reduced the growth of *Helicoverpa armigera* caterpillars (Lepidoptera) reared upon them^2^. In the decade that has followed, hundreds of studies have examined the utility of dietary RNAi against various insect pests, with widely varying success. While RNAi seems to work well in some species (e.g. some beetles and some sucking invertebrates such as mites^7^), the success is inconsistent in other species^8–11^. For example, a review of more than 150 experiments using dsRNA, found that RNAi worked much better in the family Saturniidae than in other Lepidoptera and that effectiveness depended on dsRNA concentration and the particular genes being targeted^12^.

The first publication of successful RNAi in aphids reported that microinjecting short interfering RNA’s (siRNAs) into adult pea aphids (*Acyrthrosiphon pisum*), knocked down the transcripts of *C002*, a gene that is usually highly expressed in the salivary glands^13^. Transcript levels of *C002* were significantly reduced by three days post-injection, and ultimately led to insect death by eight days, compared to 16 days for aphids injected with a control siRNA sequence. Further studies reported that *C002* is mainly expressed in a subset of cells within the salivary gland and that reduced *C002* levels prevent the aphid stylus from reaching the phloem and initiating feeding^14^. Microinjection of dsRNA into pea aphids, like siRNAs, also reduced transcript abundance of target genes *calreticulin* and *cathepsin* by 41% and 35% respectively. However, in this case there was no phenotype associated with these knock-downs^15^.

Dietary dsRNA has been delivered to aphids via artificial diet droplets placed between two sheets of parafilm^5,16^. Using this approach, pea aphids fed dsRNA homologous to *vATPase* (a gene successfully targetted in the initial beetle study^1^) exhibited ~32% reduction in transcript abundance after three days and, remarkably, achieved greater than 50% aphid mortality^5^. Another study reported that dsRNA directed to *aquaporin* via artificial diet sachets, reduced transcripts of this gene by more than 50%^17^. Although this treatment did not affect aphid weight, a reduction in the osmotic pressure of the hemolymph was observed - as expected from the knockdown of this gene.

Some strains of the green peach aphid, *Myzus persicae*, can feed on *Nicotinia benthamiana*, a plant for which transient *Agrobacterium*-mediated transformation protocols are well established. For instance, leaf discs infiltrated with *Agrobacterium* carrying vectors that express dsRNA can be fed to aphids. Two genes targeted by this approach, *C002* and *Rack1* (the latter of which encodes a gut protein) displayed 30–40% reduction in transcript levels and resulted in a moderate reduction in the number of nymphs produced by treated aphids^18^. Another study used transient transfection of *N*. *benthamiana* leaf discs to express dsRNA targeting three aphid genes (*aquaporin*, sucrase and a sugar transporter) individually and in combination and found that the combined treatment yielded a greater effect on the hemolymph osmotic pressure and body weight than the individual dsRNA treatments^19^.

Delivery of dietary dsRNA to aphids has also been achieved using plants that express dsRNA transgenes stably integrated into their genomes^12,20^. *M*. *persicae* fed on *Arabidopsis thaliana* plants expressing dsRNA directed against *C002* and *Rack1*, displayed a 50–60% knockdown of their transcripts^18^. In contrast to the earlier pea aphid microinjection study on *C002*^9^, there was no observed mortality, but there was a significant effect on aphid fecundity^18^. This fecundity effect elicited by *C002* dsRNA has been reported in a second study by the same research group but was only detected when the aphids were exposed to transformed plants over several generations^17^.

Given the apparent success of dsRNAs to induce gene knockdowns, we set out to develop dsRNA transgenes that target aphid pests of Brassica crops including cabbage, cauliflower and canola. Three closely related species of aphids - *M*. *persicae*, *Brevicoryne brassicae*, and *Lipaphis erysimi* - damage these crops directly and vector plant viruses. If these species could be controlled through plant-delivered RNAi, farmers of Brassicaceous crops might reduce their use of conventional insecticides, which are not only expensive but pollute the environment, threaten human health and have undesirable effects on pollinators. While the studies listed above identified genes that could be targeted, we initially sought novel candidates with the hope that we might elicit a greater, and therefore more effective, knockdown and hence a stronger phenotypic response. We sought genes meeting the following criteria:

1. those that were likely to elicit a strong phenotypic effect even if the knockdown was only partial (30–80% was the range of target gene knockdown reported by most previous studies),
2. those that were not too highly expressed so that targeted knockdown could have the greatest effect per unit of dietary dsRNA,
3. those that were unlikely to have a regulatory feedback mechanism that could counteract the effects of RNAi,
4. those that were expressed in tissues where RNAi was most likely to generate the strongest knockdown (i.e. the gut which is close to the site of orally delivered dsRNA entry), and
5. those that possess nucleotide sequences that enable the design of dsRNAs to particular pest species or groups.

Our initial studies focussed on (i) genes whose knockdown was known to exhibit dosage dependent phenotypes in model insects and (ii) generating a transcriptome of the *M*. *persicae* midgut so that novel targets could be identified. However, following our failure to generate phenotypes or significant transcript knockdown, we resorted to targeting genes successfully used by others. Here we report that dietary delivered dsRNAs have little or no effect on target gene abundance, even when different dsRNA sources are used (different genes produced by *in vitro* transcription or synthesized commercially) and when delivered via multiple methods (artificial diet, artificial diet with transfection reagents, transgenic *A*. *thaliana* plants) and when we assessed multiple phenotypes (including by digital PCR and quantitative real time PCR). We also report that *M*. *persicae* guts have high RNase activity and that transcripts orthologous to extracellular endonucleases of other insects are abundantly expressed in their guts.

## Results

### Preliminary screens of novel dsRNA targets showed only marginal affects on aphid weight

**Figure 1:**
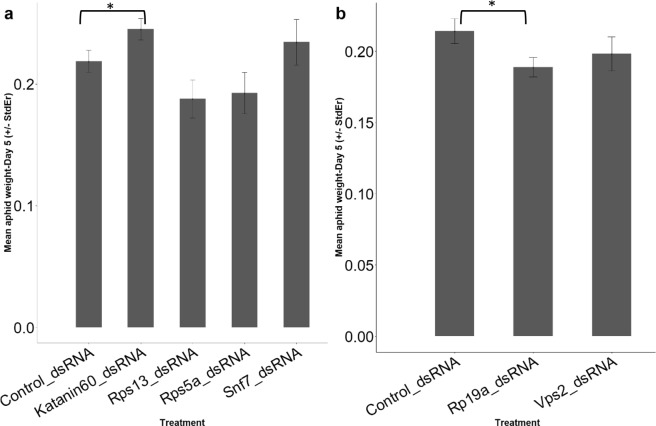
Different dsRNA fed to *M*. *persicae* in artificial diet only marginally affects aphid weight. Two experiments (**a**,**b**) were conducted at different times and targeted the following target genes: (**a**) *Rps13* (p = 0.051), *Rps5a* (p = 0.1), *Snf7* (p = 0.23), *Katanin60* (p = 0.03). Error bars show the standard error of the mean for 10 replicates. (**b**) *Vpd2* (p = 0.15), *Rp19a* (p = 0.02), asterisks shows significant change in *M*. *persicae* weight.

Given the previous reports that dietary dsRNAs may only have modest effects against aphids^16,18,20–23^, and knowing that dsRNA rarely knocks down 100% of the transcripts of the targeted gene^13,14^, we fed *M*. *persicae* various dsRNAs homologous to genes known to have dose-dependent phenotypes in other organisms. In particular, ribosomal proteins (*RpS1*3, *RpS5a*, *Rp19a*) that produce minute phenotypes in *Drosophila*, and genes involved in endosome recycling which have proven to be effective targets in beetles (*Katanin60*, *Snf7*, *Vps*2^1^). These dsRNAs were generated by first amplifying the gene from *M*. *persicae* genomic DNA using the available *M*. *persicae* sequence data to design the PCR primers and then using the PCR product for *in vitro* transcription^24^. These initial experiments used dsRNAs at a concentration of 7.5 ng/uL added to artificial diet and placed between two layers of parafilm, thereby creating sachets for aphids to feed upon. Each sachet was sufficient to maintain five aphids for at least five days. For each of these dsRNAs, we performed ten replicate experiments (i.e. total of 50 aphids per dsRNA) and then weighed the aphids. We saw no significant difference between these treatments and the negative controls in most comparisons (Fig. 1a,b). While there was a significant difference observed between the control diet and *Rp19a* in the direction consistent with an RNAi effect (Students *t*-test, p = 0.02), the other significant effect (control vs *Katanin 60*) was in the other direction (p = 0.03). We therefore sought to feed *M*. *persicae* dsRNAs homologous to those genes that have been shown to affect aphids in previous studies.

### *Mp_vATPase-like*_dsRNA fed to *M*. *persicae* in artificial diet does not affect aphid mortality or size nor does it alter target gene abundance, even when delivered with transfection agents

The *M*. *persicae* ortholog of the pea aphid *vATPase*, which was reportedly knocked down by dietary delivery of dsRNA^5^, was identified using the cited 684nt mRNA sequence (XM_00194689) as a BLAST query against the *M*. *persicae* genome^24^. This identified a gene encoding a 215-amino acid protein spread across three exons (MYZPE13164_0_v1.0_000035590.1). Phylogenetic analysis confirmed the orthologous relationship between this *M*. *persicae* sequence and the pea aphid (*Acyrthrosiphon pisum*) sequence. However it also revealed that this was a paralog not an ortholog of the *vATPase* targeted by dsRNA experiments in other species^5^ and so from hereon we refer to it as *vATPase-like* (Fig. S1).

**Figure 2:**
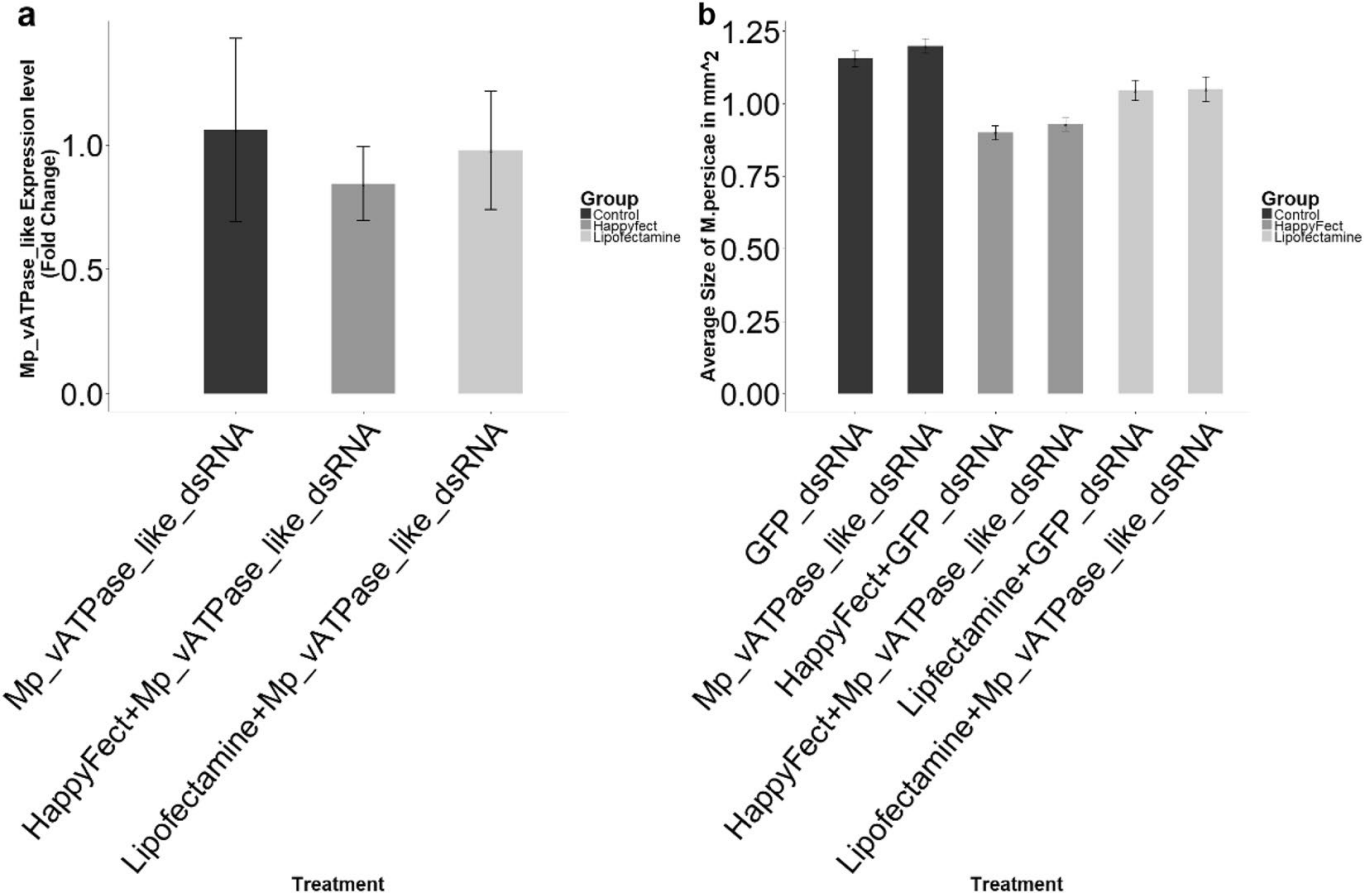
*Mp_vATPase-like_dsRNA* fed to *M*. *persicae* in artificial diet does not affect size or cause a knockdown of the target, even when delivered with transfection agents. (**a**) Quantitative real-time PCR showing that *MP_vATPase-like* transcripts level are not significantly different whether the aphids are fed *Mp_vATPase-like_dsRNA* or *GFP_dsRNA*. The fold change is not significantly different regardless of the transfection reagent used (ANOVA, p = 0.96, reps = 3). Total number of insects present in each replicate GFP_dsRNA - 4, 10, 8, Mp_vATPase – 4, 7, 8. (**b**) Aphid size does not differ between those fed on a diet containing *Mp_vATPase-like_dsRNA* and those fed *GFP_dsRNA* (Student t-test, p = 0.24), although use of the transfection agent does affect aphid size (Happyfect: p = 0.38, Lipofectamine: p = 0.94). The error bars show the standard error of the mean based on replicates = 3 replicates of 10 insects.

A 185nt *vATPase-like* dsRNA sequence, produced by *in vitro* transcription from a plasmid, was incorporated into artificial aphid diet at a final concentration of 37.5 ng/uL. After 12 days, the mortality of the aphids placed on the food, the number of offspring they produced and their size was assessed. These data were compared to a control group that had been fed 700nt dsRNA derived from a *Green Fluorescent Protein* (*GFP*) gene. No significant difference was observed between treatment and control either for insect size (p = 0.24; Fig. 2b), mortality (p = 0.39) or fecundity (p = 0.81). Furthermore, we did not detect any reduction of the *vATPase-like* transcript by quantitative reverse transcription-PCR (p = 0.96; Fig. 2a).

Transfection reagents have previously been used to improve the knockdown of target genes in insects^25–27^. We compared four commercially available transfection products. Two were lethal to aphids at the doses we used (Ribojuice^®^ and Fugene^®^). While aphids survived having Lipofectamine^®^ and Happyfect^®^ in their diets, they were marginally smaller (10–13% for the Lipofectamine^®^ treatment and 23% for the Happyfect^®^ treatment) than those without transfection reagent in the diet, irrespective of the dsRNA used. There was no additional effect on the size of the aphids resulting from the presence of the *Mp_vATPase-like* dsRNA added to their diet (Happyfect^®^: p = 0.38, Lipofectamine^®^: p = 0.94). Furthermore, an ANOVA of the *Mp-vATPase-like* transcript abundance revealed no silencing of the gene (ANOVA: p = 0.96; Fig. 2a).

### *Mp_C002_dsRNA* fed to *M*. *persicae* in the artificial diet does not knockdown the target or affect aphid fecundity even when the experiment spans two generations

We designed a dsRNA corresponding to 432 nt of the *M*. *persicae C002* gene reported in the literature^28^ and had it commercially synthesized (Genolution Inc.). Aphids were fed for 11 days on diet containing 50 ng/uL of either this dsRNA, or a negative control dsRNA (designed against *vATPase* from *Drosophila*), which had no sequence similarity to *M*. *persicae* genes. Nymphs born from these aphids were transferred to new diet sachets containing the same dsRNA and reared for another 12 days. Thus, the second-generation cohort would have developed within mothers that were reared on the dsRNA. The presence of *Mp_C002_dsRNA* did not reduce fecundity compared with the control diet, either in the first (p = 0.37) or the second (p = 0.77) generation (Fig. S2). As *C002* knockdown has been shown to contribute to mortality by disrupting the ability of aphids to find and access phloem with its stylus, perhaps phenotypic differences between treatment and control in our experiments with artificial diet in sachets would not be expected^13,18^. However, we did not detect a significant knockdown in the *Mp_C002_dsRNA* transcript expression relative to the control treatment of *Dm_vATPase_dsRNA* (Student’s *t*-test, p = 0.19, based on four replicates).

### Anti-aphid dsRNA expressed in *Arabidopsis* plants has only a minor impact on aphid numbers, even in multi-generation experiments

Ultimately, if field control of aphids is to be elicited by dietary dsRNA then it will most likely be delivered via transgenic plants. We transformed *Arabidopsis thaliana* plants of the Columbia ecotype with two transgene constructs each placed downstream of the strong CaMV *35S* promoter: (i) one designed to express exactly the same *Mp_C002_dsRNA* sequence as reported previously^28^, and (ii) one designed to express a 747nt dsRNA with sequence similarity to four *M*. *persicae* genes (hereafter called ‘BestBet’). The *BestBet* construct (Fig. S3) contains a concatemer of ~100 bp fragments from four *M*. *persicae* genes likely to elicit robust RNA silencing based on published data (148nt of *C002*^13,28^, 132nt of *vATPase-like*^5^, 154nt of *Acetylcholine esterase*^23^, and 136nt of the *snf7* ortholog^1^). Inverted copies of the combined sequence were cloned on either side of a plant intron, so that a single 400nt hairpin RNA would be produced in plants and expression of the dsRNA construct *in planta* was confirmed with qRT-PCR.

**Figure 3:**
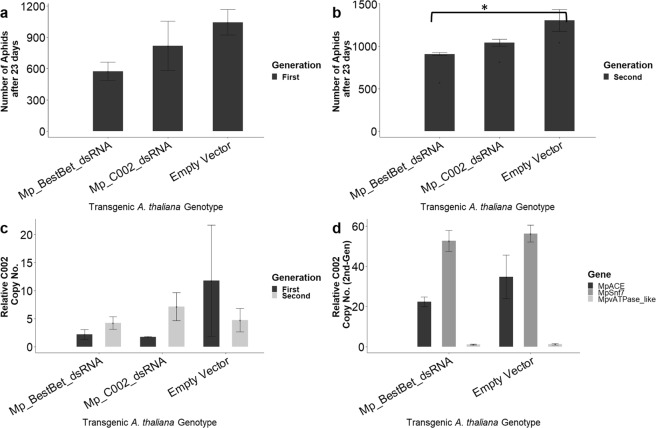
*Arabidopsis* plants expressing anti-aphid dsRNAs have only a minor impact on aphid fecundity even in multi-generation experiments. *M*. *persicae* were reared on four *Arabidopsis* genotypes: *Mp_BestBet_dsRNA*, *Mp_C002_dsRNA*, *GFP_dsRNA* and Empty vector. (**a**) The fecundity of aphids reared on these plants was measured by counting the number of aphids. No significant difference between plant genotypes was observed in the first generation. (**b**) The number of second generation aphids on the plants. One significant pairwise comparison is observed (ANOVA, p = 0.025, replicates = 3). (**c**) The level of transcript for the *MpC002* gene measured in aphids from the first and second generation using digital PCR (n = 3). Error bars are the standard error of the mean. (**d**) The level of the transcripts for *MpACE*, *MpSnf7*, *MpvATPase_like* genes was measured in aphids from the second generation using digital PCR (n = 3). Asterisks indicate significant change in *M*. *persicae* fecundity.

Ten, 1–2 day old aphid nymphs were placed onto *Arabidopsis thaliana* lines homozygous for each dsRNA-producing construct. After eleven days, ten 1–2 day old nymphs were collected from plants and transferred to fresh plants of the same genotype to start the second-generation cohort. The number of aphids present per plant were counted after 23 days of feeding for both the initial and the second-generation cohorts (Fig. 3a,b). While insect numbers on the *Mp_C002_dsRNA* plants did not differ significantly from that on the empty vector control plants in either generation, the *BestBet* plants did show a reduction in aphid numbers at a marginal significance level in the second generation (p = 0.025; Fig. 3b).

Digital PCR was then used to assess the extent of knockdown of the targeted genes. Figure 3c depicts the extent of knockdown of the *C002* gene in aphids reared for one and two generations on each of the plant genotypes. In the first generation, the variance in the ‘empty vector’ measurement in the first generation is large and no significant effect of plant genotype is observed in any of the treatments. There was no significant knockdown of *C002* observed in the second generation either. For the other three genes targeted in the *BestBet* construct, only the *ACE* gene had lower expression than the control and that difference was not statistically significant (*MpC002* − p = 0.57, *MpSnf7* − p = 0.82, *MpACE* − p = 0.39, *MpvATPase_like* − p = 0.47, n = 3; Fig. 3d).

### Ferritin satisfies the criteria as a good target gene for dsRNA yet *Mp_FeHC_dsRNA* fed to *M*. *persicae* in the artificial diet does not knockdown the target or affect aphid numbers

**Figure 4:**
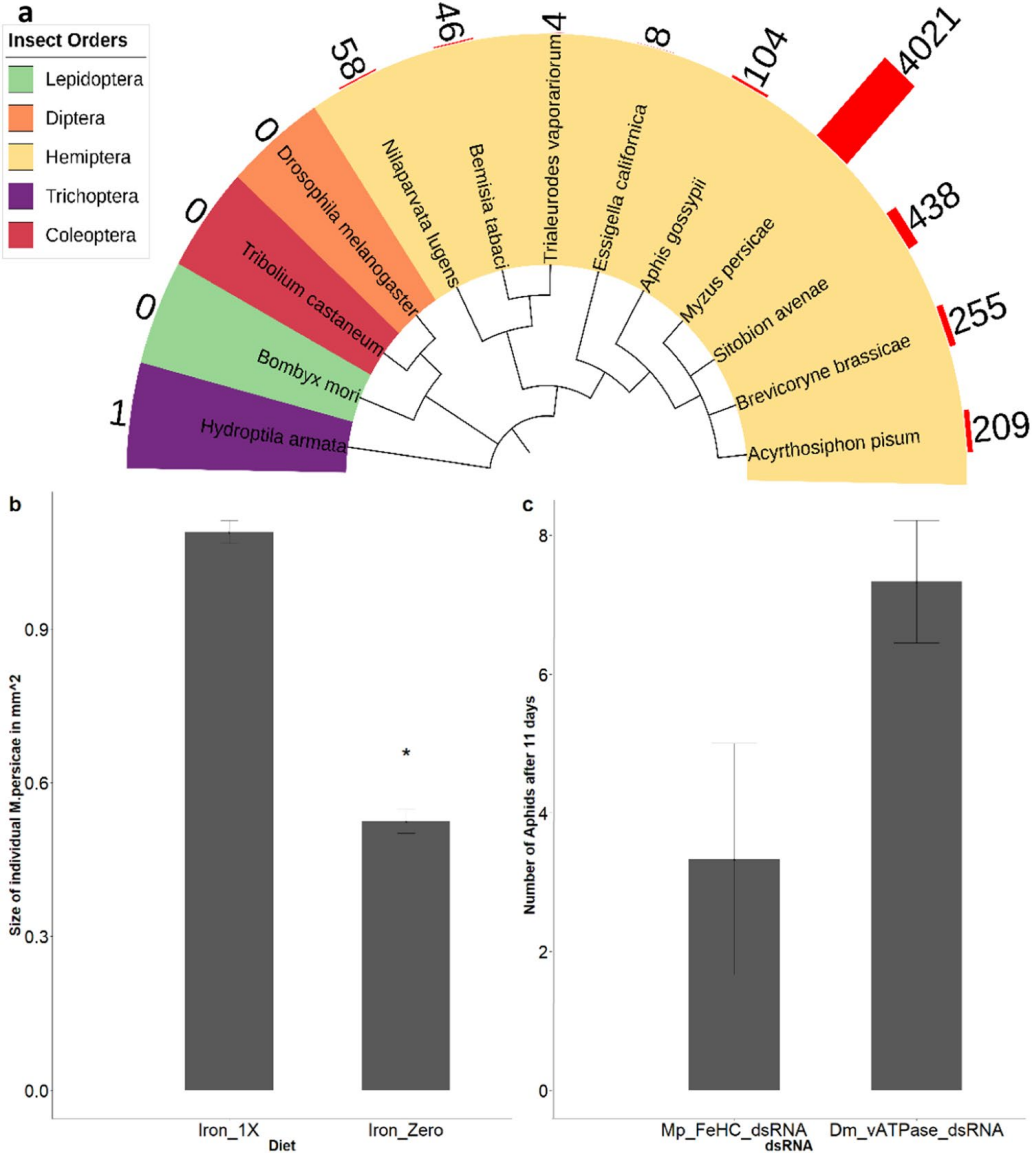
Ferritin satisfies the criteria as a good target gene for RNAi yet *Mp_FeHC_dsRNA* fed to *M*. *persicae* in artificial diet does not affect target gene activity or aphid number. (**a**) A histogram bar representing the number of 21mers matches to the 495nt of the *Mp_FeHC* gene is shown for each insect around the perimeter of the species tree cladogram (drawn based on Cytochrome oxidase I). (**b**) Dietary iron deficiency reduces aphid size (Student t-test, replicates = 3, p = 0.0001). Asterisks indicate significant differences in *M*. *persicae* size. (**c**) *Mp_FeHC_dsRNA* does not significantly affect newly born aphid numbers after 11 days of feeding relative to treatment with *Dm_vATPase_dsRNA* from *Drosophila melanogaster*. The error bars represent the standard error of the mean.

*MpC002* is a salivary gland protein, *MpACE* functions at neural synapses, *MpvATPase-like* is not well characterized and the other genes we examined are expressed broadly across tissues. We therefore sought potential novel dsRNA targets that show aphid midgut expression, as the cells of the midgut may be most accessible to dietary dsRNA and thus not require systemic spread of dsRNA following feeding. RNA-seq was performed on triplicate samples from *M*. *persicae* midguts and triplicate samples from whole aphids. An average of 29,374,419 reads was obtained from the midgut samples, and 33,489,745 reads from the whole-body samples. *De novo* transcriptome analysis was performed using the Corset pipeline^29^. The gene that attracted our attention from this analysis was that encoding the ferritin heavy chain (*Mp_FeHC*), as it was enriched in the midgut (2.4x) relative to the whole body and was distinct from the ferritin gene of non-aphid species (Fig. S4). This second point was confirmed through the application of the dsRNA taxa-specific design tool Offtarget finder^6^ which showed that there are few 21mers in the *M*. *persicae* ferritin heavy chain gene found in other invertebrates for which sequences are available, with the exception of aphids and their relatives (Fig. 4a). By these criteria, dsRNA targeting *Mp_FeHC* transcripts could easily be designed to be aphid-specific.

Another reason to focus on ferritin as an RNAi target is that aphids may be sensitive to changes in its abundance. Iron is essential and yet toxic at high doses and ferritin is thought to play a key role in its homeostasis. Whereas in mammals, ferritin is considered an iron storage protein, in insects it is believed to be involved in iron transport^30^. For *M*. *persicae*, early studies developing artificial diets revealed that trace amounts of dietary iron are indeed essential^31^. We found that if we leave iron out of the artificial diet, the aphids are half the size of those fed on standard iron-containing diet (Fig. 4b. p = 0.0001; Fig. S5) and do not reproduce. The ferritin levels in aphids does not change on diet lacking iron (p = 0.72, replicates = 3, ten aphids per replicate). Furthermore, we found that if iron levels in the artificial diet are elevated four-fold, it becomes toxic to aphids within two days.

Therefore, we tried to manipulate ferritin levels by feeding aphids commercially synthesized *Mp_FeHC_dsRNA* in an artificial diet (at a concentration of 50 ng/uL). However, no effect was observed on ferritin heavy chain transcript abundance as assessed by qRT-PCR (p = 0.52, replicates = 3) and while there was a reduction in aphid fecundity, it was not statistically significant (p = 0.10, replicates = 3, Fig. 4c).

### Transcript abundance of RNAi machinery in *M*. *persicae*

RNA-seq analysis afforded us the ability to confirm that transcripts corresponding to the RNAi machinery were present in the gut (and whole body) of the *M*. *persicae* strain used in our experiments. All the expected genes were present and most were expressed at about the same levels in the midgut as the whole body (Table 1). We include in this analysis genes implicated in the spread of dsRNA from cell to cell. While SID1 is important in the systemic spread of RNAi in *C*. *elegans*, it may not play a role in many insects either because it is not present in the genome (e.g. *Drosophila melanogaster*) or because other proteins, such as scavenger receptors associated with clathrin-dependent endocytosis may perform the function. The latter seem to take on the role in the desert locus, *Shistocerca gregaria*, which shows a strong RNAi response^32^. We note that in the *M*. *persicae* RNA-Seq that we performed, transcript abundance of the scavenger receptors are much lower in the gut relative to the rest of the body (Table 1).

**Table 1:** The RNAi machinery of *M*. *persicae*.

Gene Name	top blast hit from AphidBase transcript data	NCBI Accession	Log_2_ FC in MG	RPKM and Std Err in MG	RPKM and Std Err in WB
Dcr-1/Dcr-2	MYZPE13164_G006_v1.0_000029270.4	MN257563	−0.02	4.7 ± 0.4	4.8 ± 0.1
	MYZPE13164_G006_v1.0_000182910.3	MN257564	0.27	7.1 ± 0	5.9 ± 0.2
	MYZPE13164_G006_v1.0_000182910.2	MN257565	0.47	8.2 ± 0	5.9 ± 0.1
Sid-1	MYZPE13164_G006_v1.0_000149940.1	MN257578	0.98	16.8 ± 1.4	8.5 ± 0.3
Ago-2	MYZPE13164_G006_v1.0_000102290.1	MN257571	−0.16	31.1 ± 2.9	31.2 ± 1.7
	MYZPE13164_G006_v1.0_000150740.4	MN257572	0.66	142.4 ± 2.7	89.8 ± 0.6
Ago-3	MYZPE13164_G006_v1.0_000119300.6	MN257570	0.2	22.9 ± 1.2	19.8 ± 0.8
R2D2	MYZPE13164_G006_v1.0_000117430.1	MN257575	0.4	18.8 ± 1	14.1 ± 0.6
Aubergine	MYZPE13164_G006_v1.0_000039280.2	MN257568	−1.77	6.3 ± 0.4	21.7 ± 2.7
	MYZPE13164_G006_v1.0_000084140.1	MN257569	−5.18	0.9 ± 0.1	32.3 ± 3.8
Pasha	MYZPE13164_G006_v1.0_000186990.1	MN257567	0.03	8.9 ± 0.1	7 ± 0.1
Drosha	MYZPE13164_G006_v1.0_000018350.1	MN257579	0.2	3.2 ± 0.1	2.8 ± 0.1
Loquacius	MYZPE13164_G006_v1.0_000068140.1	MN257566	1.6	85 ± 7.3	28.1 ± 0.7
Scavenger Receptor	MYZPE13164_G006_v1.0_000124360.4	MN257558	−1.86	2.5 ± 0.1	8.9 ± 0.8
	MYZPE13164_G006_v1.0_000164000.1	MN257559	−3.91	3.2 ± 0.3	48.1 ± 7.7
	MYZPE13164_G006_v1.0_000072270.5	MN257560	−6.16	2.6 ± 2.5	6 ± 0.1
	MYZPE13164_G006_v1.0_000067150.1	MN257561	−6.83	1.2 ± 1.2	3.1 ± 0.1
	MYZPE13164_G006_v1.0_000203080.3	MN257562	−3.24	5.4 ± 3.5	18.4 ± 1.3

MG: Midgut. WB: Whole body, RPKM: Reads Per Kilobase of transcript per million mapped reads, Std Err: Standard Error, FC: Fold Change.

### dsRNase transcripts are abundant in the aphid midguts and dsRNA activity is high in midgut extracts

**Figure 5:**
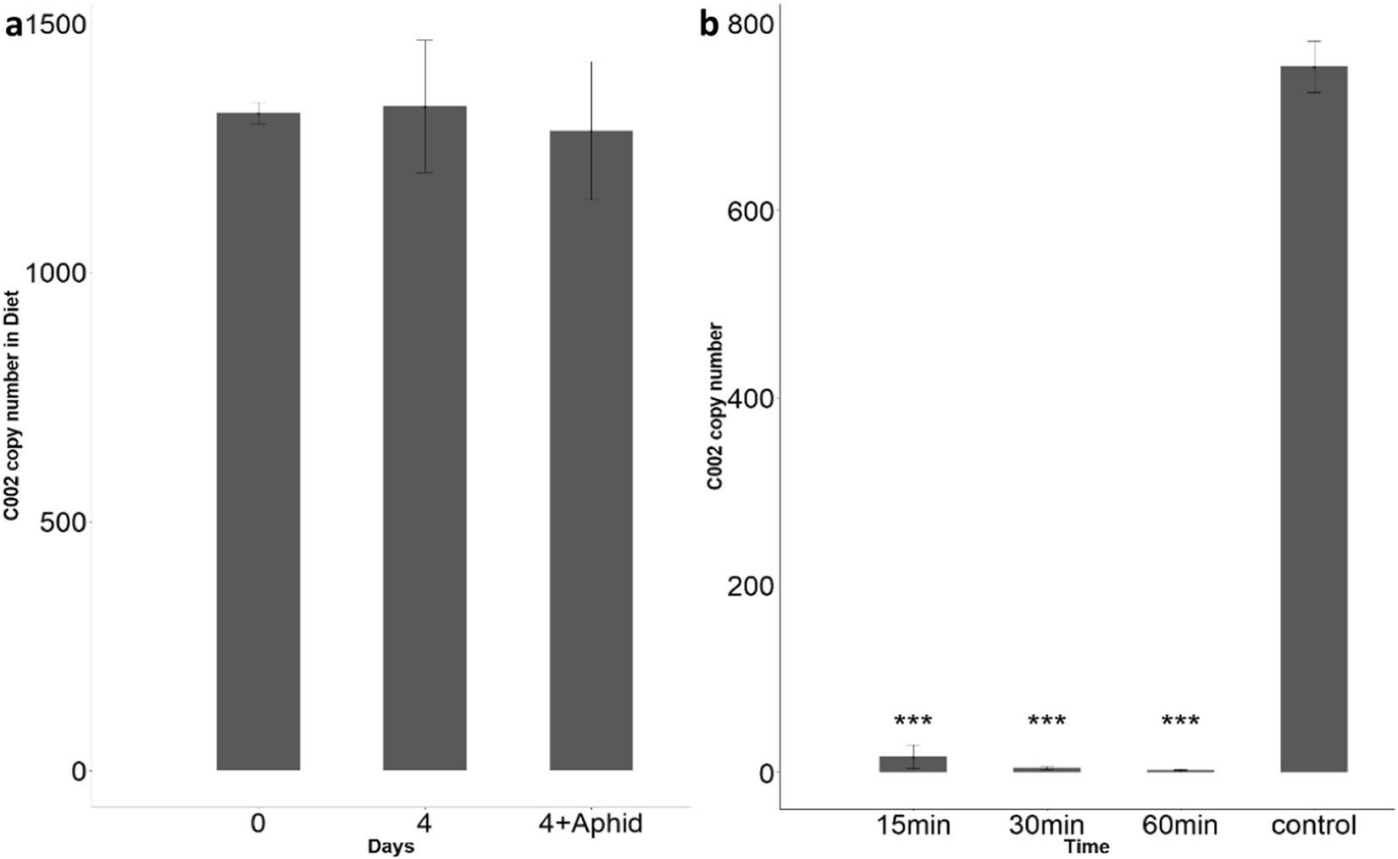
dsRNase transcripts are abundant in aphid midguts, and dsRNase activity is high in midgut extracts. (**a**) Digital PCR showing that commercially synthesized *Mp_C002_dsRNA* levels in artificial diet remain high after four days even if aphids are allowed to feed on the diet suggesting saliva is not degrading the dsRNA quickly (‘4 + adults’; ANOVA, replicates = 3, p = 0.41). (**b**) Digital PCR showing that commercially synthesized *Mp_C002_dsRNA* is rapidly degraded if incubated with aphid midguts (ANOVA, p = 0.0001, replicates = 3). The control contained dsRNA without midgut – results shown after 1 hr. Asterisks shows significant change in *MP_C002_dsRNA* concentration.

A previous study reporting the failure of RNAi treatment in pea aphids, *Acyrthrosiphon pisum*, found that aphid saliva was capable of degrading dsRNA^33^. We assessed this in *M*. *persicae* and found that there is no significant effect of saliva on the integrity of dsRNA after 4 days of incubation (Fig. 5a). However, we were motivated to examine dsRNase activity in aphid midguts. Five midguts were dissected per assay, rinsed in PBS and immediately transferred to fresh RNase-free water containing dsRNA at a concentration of 50 ng/µL. The samples were vortexed briefly and incubated at room temperature. Analysis of dsRNA concentration after 15, 30 and 60 minutes of incubations showed that the dsRNA was rapidly degraded (Fig. 5b). To characterize the speed of this degradation we repeated these experiments and found that most of the dsRNA was degraded within 5 minutes (Supplementary Fig. 8). We also found that the dsRNA degradation was inhibited by neutral saline citrate buffer (Supplementary Fig. 8) – a known nuclease inhibitor^34^.

The enzymes responsible for dsRNA degradation have been identified at the sequence level in other insects^35–37^. A search for *M*. *persicae* homologs to a *B*. *mori* endonuclease that has been shown to degrade double stranded RNA, identified three transcript clusters with high identity, each of which is highly abundant in the midgut and enriched in the midgut relative to the whole-body samples (Table 2). These map to a single gene in the *M*. *persicae* genome (official *Myzus* assembly ID - MYZPE13164_G006_v1.0_000023850.1). This gene is therefore a strong candidate to encode an RNase enzyme that is capable of degrading dietary dsRNA.

**Table 2:** Expression level of dsRNases in the midgut (MG) and whole body (WB) of *M*. *persicae*.

Gene Name	top blast hit from AphidBase transcript data	NCBI Accession	Log_2_ Fold change in Gut	RPKM and Std Err in MG	RPKM and Std Err in WB
dsRNase	MYZPE13164_G006_v1.0_000023850.1	MN257276	4.13	526.4 ± 65.6	30 ± 1.2
	MYZPE13164_G006_v1.0_000023850.1	Cluster-21088.2 (probable isoform)	4.1	523.5 ± 62.7	30.5 ± 1.8
	MYZPE13164_G006_v1.0_000023850.1	Cluster-21088.3 (probable isoform)	4.1	595.1 ± 68.8	34.6 ± 1.5

RPKM: Reads Per Kilobase of transcript per million mapped reads, Std Err: Standard Error, FC: Fold Change.

**Figure 6:**
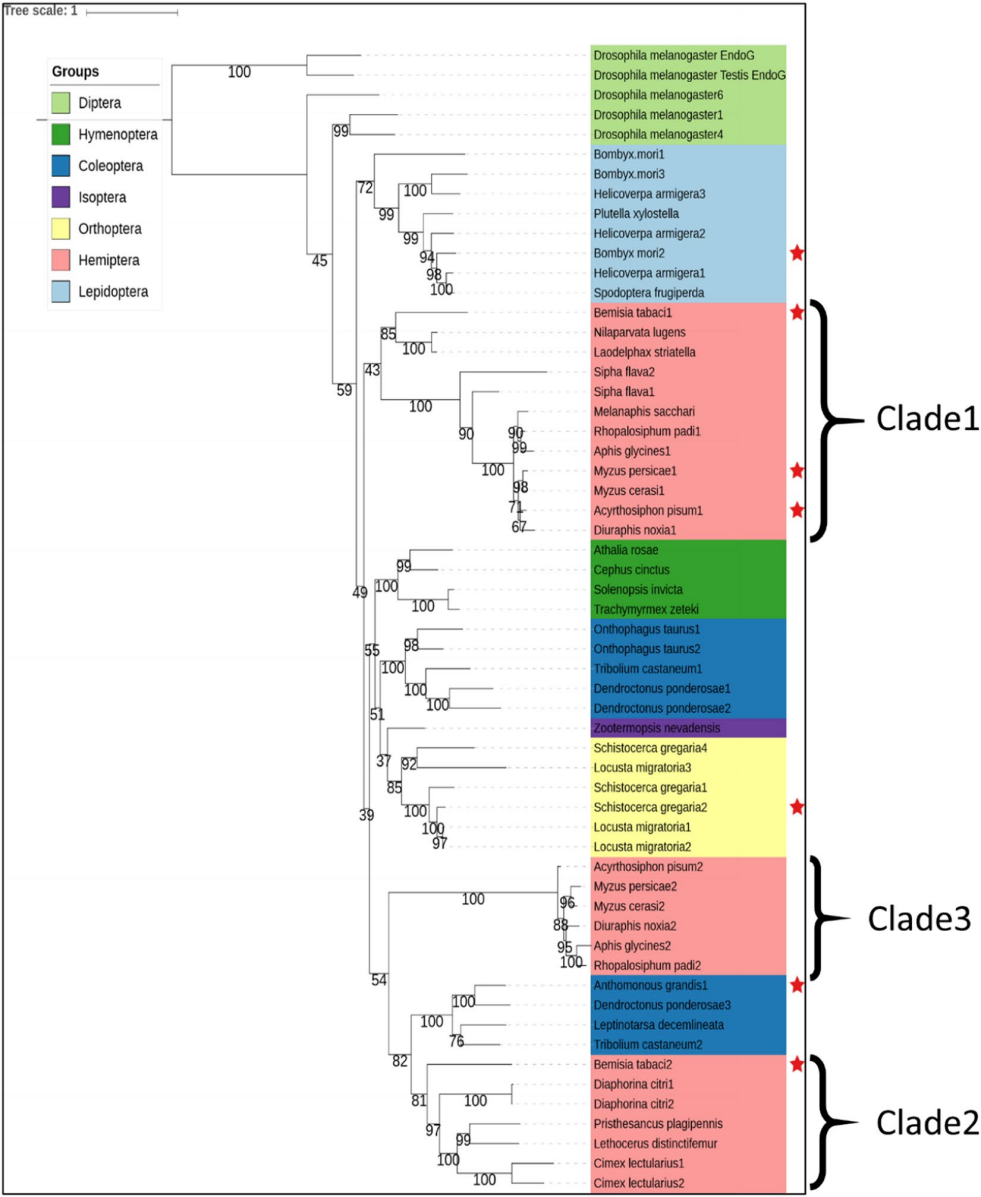
Extracellular Endonucleases (IPR20821) of insects. A maximum likelihood phylogenetic tree of a subset of insect extracellular endonucleases. Bootstrap scores are shown as percentages. The asterisks represent sequences that have dsRNA activity reported in the literature. The endo-G clade was specified as an outgroup.

**Figure 7:**
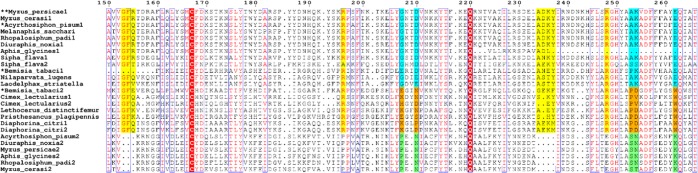
Alignment of three independent hemipteran clades. Sequence features distinctive to clade 1 endonucleases are shown in cyan, those for clade 3 are shown in orange and those for clade 2 are shown in light green. Deletions in clade 2 are shown with yellow colour highlights. Red colour highlights show conserved residues. Red colored residues have similar chemical properties.

To examine this possibility more thoroughly we performed phylogenetic analyses on 144 insect and crustacean amino acid sequences that have been classified in Interpro family IPR020821 (extracellular endonucleases) or that are homologous to these sequences. This included six sequences that have been shown to have dsRNA activity. In general, the phylogeny shows high-confidence clades can be assigned to insect orders (Supplementary Fig. 7). There is a lepidopteran clade, an orthopteran clade, a hymenopteran clade and a clade containing termite and cockroach sequences. There are multiple independent dipteran clades and two independent coleopteran clades and, most pertinently, three independent hemipteran clades. One of the three hemipteran clades contains an aphid clade nested within a broader hemipteran clade (clade 1 of Fig. 6), another consists of aphid sequences only (clade 3), and the third consists of non-aphid sequences (clade 2). Various sequence motifs in the alignment help define these clades including an insertion near the C-terminus of clade 1 sequences, and small deletions in the clade 3 sequences (Fig. 7).

Recently, inhibition of a nuclease-encoding gene was shown to enhance RNA interference in the closely related aphid *Acyrthrosiphon pisum*^38^. Our phylogenetic analysis clearly shows that the *Acyrthrosiphon pisum* and the midgut-expressed *Myzus persicae* sequences described above, are orthologous (clade 1, **Myzus_persicae1). A sequence from the whitefly, *Bemisia tabaci*, also shown to have RNase activity, is located within in this clade, although the sequence accession (AQU43106) shows it has a four hundred amino acid N-terminal extension that is quite distinct from other endonuclease sequences. The clustering of these sequences into a single clade (clade 1) is consistent with the *M*. *persicae* sequence encoding a midgut nuclease capable of digesting dsRNA, especially since it shares 82% amino acid identity with the *A*. *pisum* sequence.

## Discussion

In contrast to a previous study on pea aphids (*Acyrthrosiphon pisum*) we were unable to elicit gene knockdown by feeding aphids *vATPase-like* dsRNA in an artificial diet, even though we were using higher concentrations of dsRNA (20 ng/µl in the grain aphid^39^ versus 37.5 ng/µl in the present study). We were unable to elicit an effect by supplementing the dsRNA with various transfection reagents. Our experiments were not a strict replication of the pea aphid study because the aphid species are different. So, it is possible that ineffectiveness of dsRNA towards *M*. *persicae* might reflect species differences. The function of *vATPase-like*, is not well understood (in contrast to its paralog vATPase), but it is expressed at low levels in the whole body and gut transcriptome datasets that we generated and confirmed by qRT-PCR and digital PCR assays. It is possible that its role is more critical in *A*. *pisum* than *M*. *persicae*.

The *C002* gene, which encodes a salivary protein, has been targeted by dsRNA in both *A*. *pisum* and *M*. *persicae*. In *A*. *pisum*, injected siRNA interferes with the ability of the aphids to feed on plants and consequently results in mortality^13^. In *M*. *persicae*, *in planta* delivery of dsRNA against *C002* did not impact upon mortality, but was reported to significantly reduce reproductive output^18^. The impact on fecundity has been reported in two studies from the same research group, with the second study observing an impact only in a multigenerational study^28^. Thus, it has been attributed to a transgenerational effect where nymphs, born to aphids fed upon dsRNA-expressing plants were affected by maternally ingested dsRNA^28^. In the experiments described here, *Mp_C002_dsRNA* delivered through artificial diet did not affect reproductive output even when it spanned two generations of feeding. We also saw no decrease in reproductive output of aphids reared for two generations on transgenic *A*. *thaliana* plants (selected to produce high *Mp_C002_dsRNA* levels) relative to control plants of the same genetic background. We also did not see significant knockdown of *C002* transcripts in aphids reared on these plants, even when we assessed this by the potentially highly accurate digital PCR technique.

We did observe that feeding on our multigene composite dsRNA ‘*BestBet*’ plants did produce significantly reduced numbers of aphids relative to those fed on ‘empty vector’ control plants, at a marginal level (p = 0.025). Given the number of statistical tests performed this may be a type I error (occasionally we expect a number less than the significance threshold even if the null hypothesis is true). Our analysis of the transcripts of the four genes targeted by this construct revealed that those from *MpACE*, which encodes acetylcholine esterase, was the only one with reduced abundance, albeit not significantly so.

We also selected a gut enriched target gene for RNAi study. Such a gene should give dietry dsRNA access to the target gene immediately after entering the digestive system. The midgut specific ferritin heavy chain transcript was selected from the tissue transcriptome study based on the five selection criteria described in the Introduction. In *D*. *melanogaster*, knockdown of ferritin resulted in iron deficiency, iron accumulation in the gut and neuronal damage^40^. The artificially synthesized *Mp_FeHC_dsRNA* did not show any effect on *M*. *persicae* survival, size, fecundity or expression level of the *Mp_FeHC* suggesting that dsRNA-mediated gene silencing does not elicit a silencing response within the gut tissue or digestive system of *M*. *persicae*.

In general, the evidence that dietary delivered dsRNA can elicit a robust response in *M*. *persicae* is far from compelling in our experimental analyses. We have shown that transcripts for all the proteins known to be required for RNAi to work are expressed in the midguts of *M*. *persicae*, although the scavenger receptors that may play a role in the systemic spreading of dsRNA in other organisms, such as the desert locust^31^, are reduced in the gut of *M. persicae* relative to the whole body.

An explanation for the failure of dsRNA to elicit knockdown effects comes in the abundance of transcripts encoding putative dsRNases in the gut of the aphids we have studied. We demonstrated that dsRNA incubated with aphid guts is quickly degraded and that the degradation is inhibited by standard saline citrate - a known RNase inhibitor (Fig. S8)^34^. Degradation of dsRNA has also been observed in the desert locust, where injected dsRNA reportedly works robustly, but dsRNA delivered by feeding is ineffective^41^. Furthermore, it has recently been reported that dsRNA was completely degraded when incubated with midgut homogenate of the cotton boll weevil (*Anthonomous grandis*)^42^ and in another weevil a dsRNase gene that appears to have a signal peptide and is highly transcribed in the gut was shown to degrade dietary RNA^43^. However a recent publication^38^ has the pertinent information that a knockdown of the the ortholog of the putative dsRNase we identified (*nuc1*) enables RNA interference in the closely related aphid species, *Acyrthosiphon pisum*. There are now examples of moth, locust, whitefly, beetle and aphid dsRNases inhibiting RNAi^35,36^. All belong to the same interpro family, and support the proposition that the *M*. *persicae* clade 1 version is responsible for the rapid disappearance of dsRNA when incubated with the gut.

How then do we interpret the results of previous studies showing effective RNAi in aphids? If gut RNases are degrading dsRNA, there is no issue with respect to the microinjection studies because dsRNA is not exposed to the gut dsRNases. The feeding studies, via artificial diet, or *in planta*, are less easy to explain. Work on another species of locust (*Locusta migratoria*) may provide an answer. It was recently reported that geographically defined strains of locust differed in their susceptibility to dsRNA mediated RNAi^44^. Furthermore, by crossing different strains the authors present evidence that the variation in susceptibility has a genetic basis and that the resistant form was probably dominant to the susceptible forms. So, it is possible that the *M*. *persicae* strains used in this study (Bona vista and c61), which were collected in Australia, have genetic variants that make them more resistant to RNAi than *M*. *persicae* strains used by other researchers. Another argument for strain to strain variation was posed by Swevers *et al*.^45^ who suggest that some insects could harbour viruses that interfere with the RNAi process. Variation in dsRNA susceptibility between aphid strains therefore warrants further study for two reasons. Firstly, an understanding of such variation may suggest strategies to make RNAi generally effective. Secondly, if there are some strains of *M*. *persicae* that are susceptible to dsRNA then there is still the possibility that RNAi could be used for functional genomics studies, a strategy that could be enhanced by exploiting the ‘priming’ phenomena recently described in pea aphids^46^.

Finally, what are the prospects for using dsRNA technology to control aphid populations? Chung, *et al*.^38^ found that if they co-administered dsRNA directed against the endonuclease 1 gene (*nuc1*) with their desired target gene then RNA interference was effective. Such a co-administration strategy could be applicable to field situations. Alternatively, recent work suggests that there is some hope that dsRNA can be protected from RNases with a protein^42^ or by a guanylated polymer^47^ which may also help transport it into insect cells. Alternatively transgenic plants that have ribonucleoprotein particles (consisting of dsRNA and these proteins that protect and direct dsRNA) would need to be directed to the phloem of plants in a way that does not interfere with normal physiological processes. Or, perhaps there are prospects for ribonucleoprotein particle sprays that could be absorbed by the aphid cuticle and thereby minimize the effect of midgut RNases altogether. Recent work shows that topical application of dsRNA can knockdown genes in various aphid species, even at doses as low as 60 ng, and so there is still hope that dsRNA based biopesticides might be effective^48^.

## Methods

### Insect rearing

Apart from the initial experiments reported in Fig. 1a,b, which used the C61 strain, the experiments were performed on a *M*. *persicae* strain collected by Dr. Paul Umina from Bona Vista Rd Warragul, VIC, AU (38°13′01.6″S 145°58′19.5″E; Collection date: 22/03/2012, Host plant: *Raphanus raphanistrum*). Aphids were maintained at 20 °C with 12 h/12 h, dark/light period on Radish plants (*Raphanus sativus*). At the start of these experiments, the aphid colony was established from a single aphid to avoid unwanted natural diversity within the experimental population of this clonal species.

### NextGen sequencing and new target selection

After feeding on the dsRNA-containing diet, dsRNAs enter the digestive system of the aphid. This motivated us to identify genes that are differentially expressed in the midgut (MG) of the insect as compared to the whole body (WB). The midgut of about 1000 *M*. *persicae* (2^nd^ and 3^rd^ instar) were dissected out over several days. Dissected guts were immediately transferred to fresh ice-cold Trizol® solution and stored at −80 °C until further processing. On the day of RNA isolation, dissected samples were randomly combined into three pools and these represent biological replicates. Similarly, three groups of ten 2^nd^ and 3^rd^ instar, whole body *M*. *persicae* were placed in separate tubes. RNA isolation was performed using a DirectZol® RNA isolation kit. The quality of isolated RNA was checked on an agarose gel and quantified using the Qbit™ system. A 3 µg/sample of RNA was supplied to the Australian Genome Research Facility (AGRF) company for library preparation using poly-A selection. The sample libraries were pooled and sequenced on HiSeq 2500 system with 100 bp read length.

Differential gene expression analysis was performed using the standard EdgeR analysis pipeline using the following software – FastQC, Trimmomatic, STAR and EdgeR. FastQC and EdgeR was used with default settings. Supplementary Data-3 outlines the modified commands used for STAR and Trimmomatic. A list of genes was generated showing differentially expressed transcripts in the midgut of *M*. *persicae* compared to whole-body samples.

### Effect of Iron (Fe) on development of *M*. *persicae*

Artificial aphid diet^49,50^ containing three different concentrations of iron (*FeCl*_*3*_.*6H*_*2*_*O*) were fed to *M*. *persicae*: no iron in the diet, a normal diet with the recommended iron concentration, and four times more iron in the diet. Three replicates of ten, 1–2 day old, nymphs were fed on the respective diets for 12 days. The insects were observed for phenotypic changes, fecundity and mortality.

### dsRNA preparation

dsRNA was generated using two different methods. The *M*. *persicae* orthologs of *Drosophila melanogaster Rps13*, *Rps5a*, *Snf7*, *Katanin60*, *Vpd2*, and *Rp19a* were identified by BLAST against the available databases and specific PCR amplicons were designed and amplified. dsRNAs from these sequences and from *dsMPvATPase-like* and *dsGFP* were synthesized artificially for diet incorporation using the MEGAscript® T7 *in vitro* transcription kit (Ambion®) for artificial diet incorporation. The *MP_vATPase-like_dsRNA* was 185 bp long. It was generated using PCR with oligonucleotide primers (Supplementary Data 1).

*Mp_C002_dsRNA*, *Dm_vATPase_dsRNA* and *Mp_FeHC_dsRNA* were artificially synthesized by Genolution, Inc. to ensure that any effects, or lack of them, could not be attributed to contaminants which may be present in *in vitro* transcribed dsRNA. The sequence for *C002* dsRNA of *M*. *persicae* (*Mp_C002_dsRNA*) was obtained from Coleman *et al*.^28^. For the artificial synthesis of *Mp_C002_dsRNA*, the fragment length was 496 bp, deleting 64 bp from the start and 150 bp from the end. *Drosophila melanogaster vATPase* dsRNA (*Dm_vATPase_dsRNA*) was synthesized as a negative control.

### Artificial diet bioassay

Artificial diet bioassays for *Rps13*, *Rps5a*, *Snf7*, *Katanin60*, *Vpd2* and *Rp19a* were carried out over 5 days. dsRNA of each candidate gene was fed via artificial diet at a concentration of 7.5 ng/µl. There were 10 replicates of each treatment with 5 insects in replicate. Mortality of insects was recorded on day 3 and day 5. On day 5, all the live insects from each replicate were weighed together on a microbalance.

Artificial diet bioassays were carried out using *Mp_vATPase_dsRNA* and *Mp_C002_dsRNA* to explore their potential to affect the fitness of *M*. *persicae*. The final concentration of dsRNA in the diet was 37.5 ng/µl for *Mp_vATPase_dsRNA* and 50 ng/µl for *Mp_C002_dsRNA* and *Mp_FeHC_dsRNA*. *GFP_dsRNA* (37.5 ng/µl) and *Dm_vATPase_dsRNA* (50 ng/µl) were used as negative controls for *Mp_vATPase_dsRNA* and *Mp_C002_dsRNA* respectively.

Clear acrylic pipes (25 mm × 35 mm) open at both ends were used as cages to perform the bioassay. One side of the cage was closed by stretching two layers of parafilm over it. A group of ten, 1–2 day old, *M*. *persicae* nymphs were carefully transferred into the cage using a paint brush. The other end of the cage was sealed with parafilm layers containing diet with/without dsRNA. All the cages were incubated at 20 °C with 12/12 hr day/night photoperiod and aphids were transferred to fresh satchets every four days. Observations on the survival and fecundity of *M*. *persicae* were recorded after 12 days.

To identify any transgenerational effects of *Mp_C002_dsRNA*, *M*. *persicae* were monitored for 12 days (with three changes of diet sachets) in the cages with/without diets containing dsRNA. All the newborn nymphs were then transferred to new cages with fresh diet of the same type as used in the previous generation. Every treatment was repeated three times (i.e. replicates = 3). The final number of aphids was counted after a further 12 days.

We also tested transfection reagents (Happyfect® by Tecrea, Fugene® by Promega, Ribojuice® by Millipore and Lipofectamine®by Thermo Fisher Scientific) for their potential to enhance the delivery of dsRNA via artificial diet. Toxicity of the transfection reagent was determined by feeding 2 µl of transfection reagent mixed in 100 µl diet and fed to aphids for 12 days. During all the artificial diet bioassays, the diet was changed every four days or when bacterial or fungal contamination was found in the diet if this was earlier than four days.

### *M*. *persicae* size analysis

Aphid fecundity and weight are directly proportional to size^51^. We developed a macro script for ImageJ software that enables determination of the size of the insect in mm^2^ (Supplementary Data-4).

### Relative gene expression analysis of samples from the artificial diet experiment

*M*. *persicae* samples were collected from all the treatments at the end of the bioassay for each generation. The insects were separated based on their morphology (alate or wingless), and morphologically similar insects from each replicate were pooled. Only wingless insects were carried forward for expression analysis. *Mp_C002_dsRNA* treated replicates had 10, 10, 10, 9, 6 insects in five biological replicates whereas *Dm_vATPase_dsRNA*-treated replicates had 10, 9, 9, 9, 6 insects. Total RNA was extracted from each sample using a Direct-zol™ RNA kit. The first strand of cDNA was synthesized using MuMLV reverse transcriptase (NEB) according to the manufacturer’s instructions. Gene expression analysis was then performed for each sample using real-time PCR analysis. The ∆∆ct method of relative quantification determined the difference in expression of target genes in test and control samples^52^. The primers for five different housekeeping reference genes (*GDPH*, *RpL7 RpS3*, *Actin*, *Tubulin*; Supplementary Data-2) were tested for their efficiency and stable expression. *RpL7* was selected based on its stable expression and primer efficiency (Slope = 1.9).

### Development of transgenic Arabidopsis expressing dsRNA

The *Mp_C002_dsRNA* fragment was designed using primers described by *Coleman et al*.^28^. The *Mp_C002_dsRNA* fragment of 710 bp was amplified and cloned into a RNA hairpin producing vector, pL4440, then sub-cloned into the binary vector pMLBART (Fig. S3).

The multigene ‘Bestbet’ dsRNA construct was created by concatenating the sequences of four genes which had orthologs shown to elicit a dsRNA response in the literature: *Snf7*^1^, *vATPase-like*^5^,*C002*^13,28,39^, *AChE*^23^; sequence available in Supp. Data-1. This was synthesized by Biomatik with specific restriction sites at both ends. The synthesized sequence was initially cloned into a RNA hairpin-producing vector, pL4440 then sub-cloned into pMLBART (Supplementary Fig. 3).

*Mp_C002_dsRNA* and *Mp_BestBet_dsRNA* constructs were introduced into *Agrobacterium tumefaciens* strain C58 and then used to transform *A*. *thaliana* plants (Col-0 ecotype) using the floral dip method^53^. Seeds from the dipped plants were sown in potting soil and germinating seedlings were sprayed with phosphonocithrin (Basta^®^: Bayer) to select transformants. Lines were established from these individual plants by letting them self-pollinate. Seeds from each of these transformed plants (T1) were harvested, sown and seedlings also exposed to BASTA screening. Lines displaying a ratio 3:1 Basta resistant/Basta sensitive were likely to possess a single transgene insertion. Basta resistant individuals were subsequently screened for homozygosity in the next generation. This screening procedure resulted in ten *Mp_C002_dsRNA* and six *Mp_BestBet_dsRNA* lines, that were confirmed to have the transgene by PCR using insert-specific primers and subsequently screened for expression levels of dsRNA at the seedling stage (Fig S6, Supplementary data 2). The three highest expressing lines from each construct were selected for further analysis.

All transgenic and non-transgenic *Arabidopsis thaliana* plants were maintained at 20 °C under a 24 h photoperiod.

### Transgenic plant bioassay

The selected *A*. *thaliana* plants were sown individually in small cups. After four weeks of growth, a group of ten 1–2-day old *M*. *persicae* nymphs were released on each plant. Every plant was then caged in a separate plastic box and maintained at 20 °C and 12/12 hr, day/night photoperiod. This group of treatments were regarded as the first generation. On the 10^th^–11^th^ day from the start of the experiment, the aphids started reproducing new nymphs. A group of newly born, 1–2 day old, nymphs were then released onto a fresh plant of the same genotype. This group of treatments were regarded as the second generation. Plants harbouring insects of one or other of the generations were maintained in plastic containers for 23 days. Then the number of aphids on each plant were counted and represented the fecundity measure. A parallel set of plants were set up for both generations that enabled ten insects to be harvested on the fourth day from the start of the experiment, for RNA isolation and digital PCR analysis.

### Effect of *M*. *persicae* saliva on dsRNA stability in artificial diet

The effect of *M*. *persicae* saliva was analysed by feeding insects on a diet containing *Mp_C002_dsRNA* with 50 ng/µl of diet. A diet with *Mp_C002_dsRNA* was also maintained under the same conditions but without insects to observe diet-dsRNA interaction and its effect on dsRNA stability. dsRNA + diet was incubated with/without insects for 2 days and for 4 days. Diet samples were used for cDNA synthesis and digital PCR to quantify the number of dsRNA copies present in the solution. 0.16 M of standard saline citrate was used in inhibition assays.

### Effect of dissected gut tissues on dsRNA stability

To examine dsRNA degradation in gut tissue, five midguts were dissected from 2^nd^ and 3^rd^ instar *M*. *persicae*. The dissected guts were washed with PBS and immediately transferred in a fresh RNase free water containing dsRNA at a concentration of 50 ng/µl. The samples were vortexed briefly and incubated at room temperature. Subsamples were collected after 15 min, 30 min and 60 min of incubation and stored at −80 °C. A sample with no midgut was used as an experimental control. The collected samples were used for cDNA synthesis and digital PCR for absolute quantification of dsRNA copies in the sample.

The dsRNA stability was also checked in the presence of the endonuclease inhibitor, neutral saline citrate buffer. dsRNA (50 ng/µl) was incubated with dissected midgut of *M*. *persicae* for 1 min, 5 min and 10 min and also incubated with neutral saline citrate buffer for 15 min. Samples were visualized using polyacrylamide gel electrophoresis.

### Phylogenetic analysis

To assess the phylogenetic relationship of the putative nuclease from *Myzus persicae* we assembled related sequences from the literature^54^, from databases (Extracellular Endonucleases subunit A from Interpro IPR020821 and Pfam 01223), and from Blast searches of NCBI protein and nucleotide databases. Furthermore, we performed tblastn searches of the accessible aphid sequences on Aphidbase (https://bipaa.genouest.org/is/aphidbase/) and manually annotated related sequences using Artemis software^55^. The sequences were curated to remove repeated entries, partial sequences (except for those in the hemipteran clade) and to split multiple-nuclease-domain proteins into component parts. This involved an iterative process of sequence inclusion/exclusion, alignment, and tree building. For the final phylogenetic analyses the multiple sequence alignment was performed with MAFFT software^56^. Phylogenetic tree building was performed using the W-IQ-tree online tool using its default settings. W-IQ-tree tests 168 different substitution models using a model finder algorithm to find the best-fit model which was WAG + I + G4. The tree space was then explored using the nearest neighbour interchange (NNI) algorithm. The phylogenetic tree was then visualised using iTOL online tree viewer.

### Accession codes

RNA-Seq data is lodged in NCBI’s SRA database with accession number PRJNA556546.

## Supplementary information

Supplementary Information
