# Supplementary material for: Extracellular endonucleases in the midgut of Myzus persicae may limit the efficacy of orally delivered RNAi
Source: Sci Rep. 2019 Aug 15;9:11898. doi: 10.1038/s41598-019-47357-4 (PMC6695413; doi:10.1038/s41598-019-47357-4)
Supplement: Supplementary file 1 — Supplementary Information [file 41598_2019_47357_MOESM1_ESM.pdf]

Title:

Extracellular endonucleases in the midgut of *Myzus persicae* may limit the efficacy of orally delivered RNAi

Authors: Amol Bharat Ghodke[1], Robert Trygve Good[1], John Golz[1], Derek A. Russell[2], Owain Edwards[3], Charles Robin[1]*

1. School of BioSciences, The University of Melbourne, Australia
2. Faculty of Veterinary and Agricultural Sciences, The University of Melbourne, Australia
3. Ecosystems Science, CSIRO, Perth, Australia

* Corresponding author

Supplementary material:

Supplementary Figure 1: Phylogeny of vATPase and vATPase-like

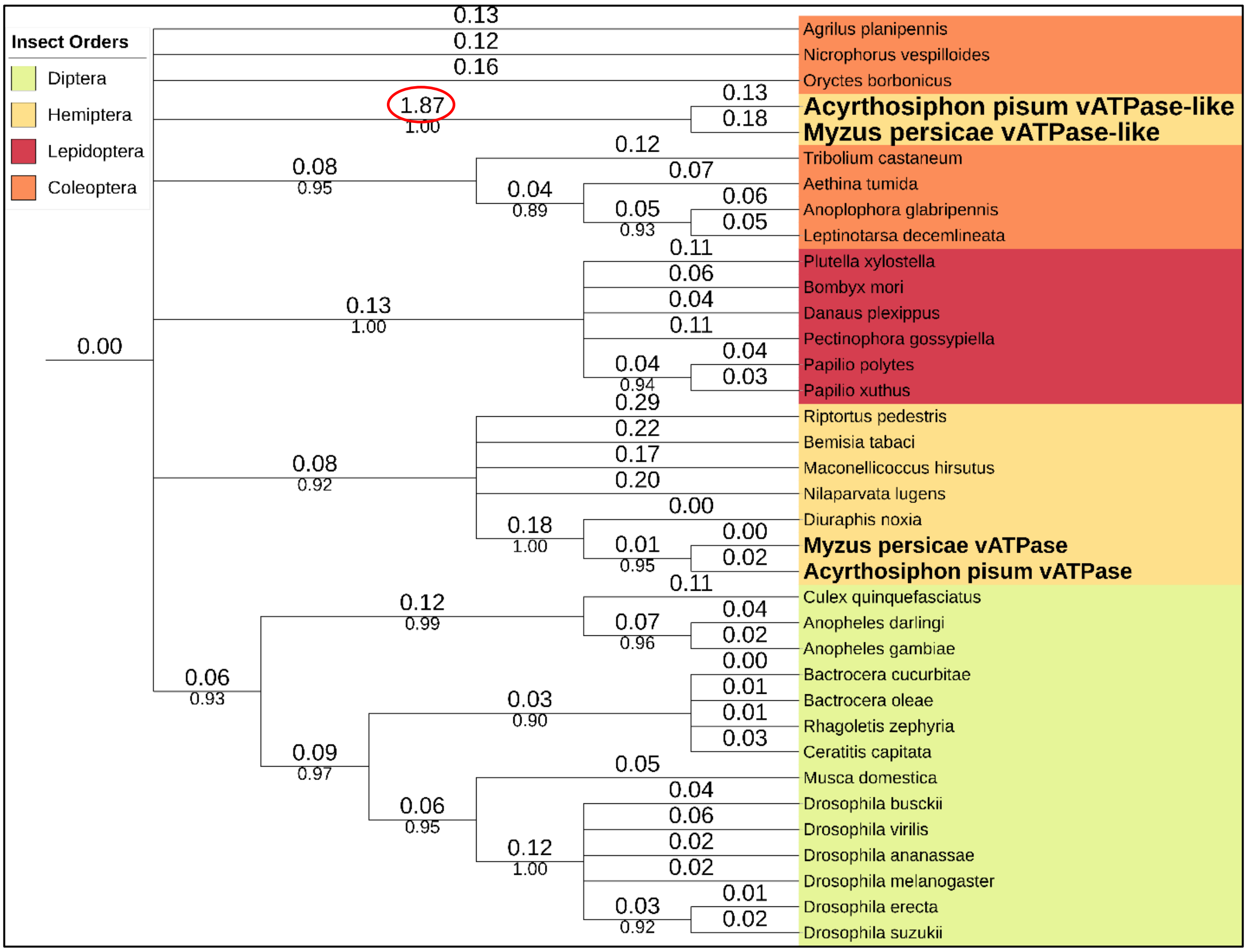

Supplementary Figure 1 – The aphid genome encodes vATPase and vATPase-like genes. The phylogenetic tree suggests these two genes diverged before hemipteran species radiated. The numbers in small font shown below the branches represent the proportion of bootstrap replicates that support that branch and the numbers in large font above the branches indicate branch lengths. The branch length of the *MP_vATPase-like* sequence (circled) is long compared to the real *vATPase* sequences from a diverse range of insects.

Supplementary Figure 2: ***Mp_C002_dsRNA*** **feeding assay**

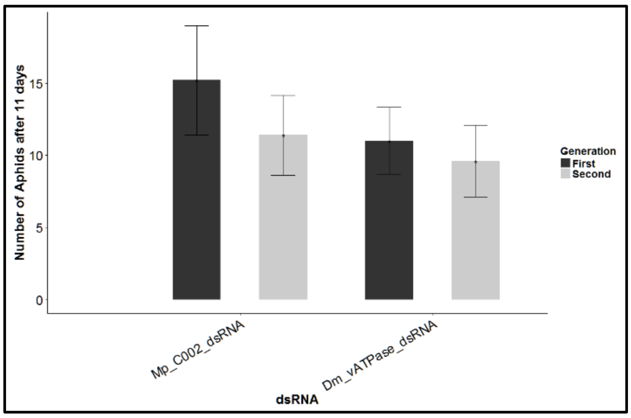

Supplementary Figure 2: ***Mp_C002_dsRNA*** **fed to** ***Myzus persicae*** **in the artificial diet does not knockdown the target or affect aphid fecundity even when the experiment spans two generations.** Commercially synthesized *Mp_C002_dsRNA* was fed to 5 replicates of 10 - aphids for 11 days and the number of progenies was recorded. dsRNA sequence derived from *Drosophila melanogaster vATPase* was used as a negative control. For each replicate, 10 of the progenies were then transferred to fresh media, and the number of their progeny produced was recorded (Student *t*-test, first generation - P=0.37, Second Generation - P=0.77) The error bars represent the standard error of the mean.

Supplementary Figure 3: Constructs used in Arabidopsis transformations

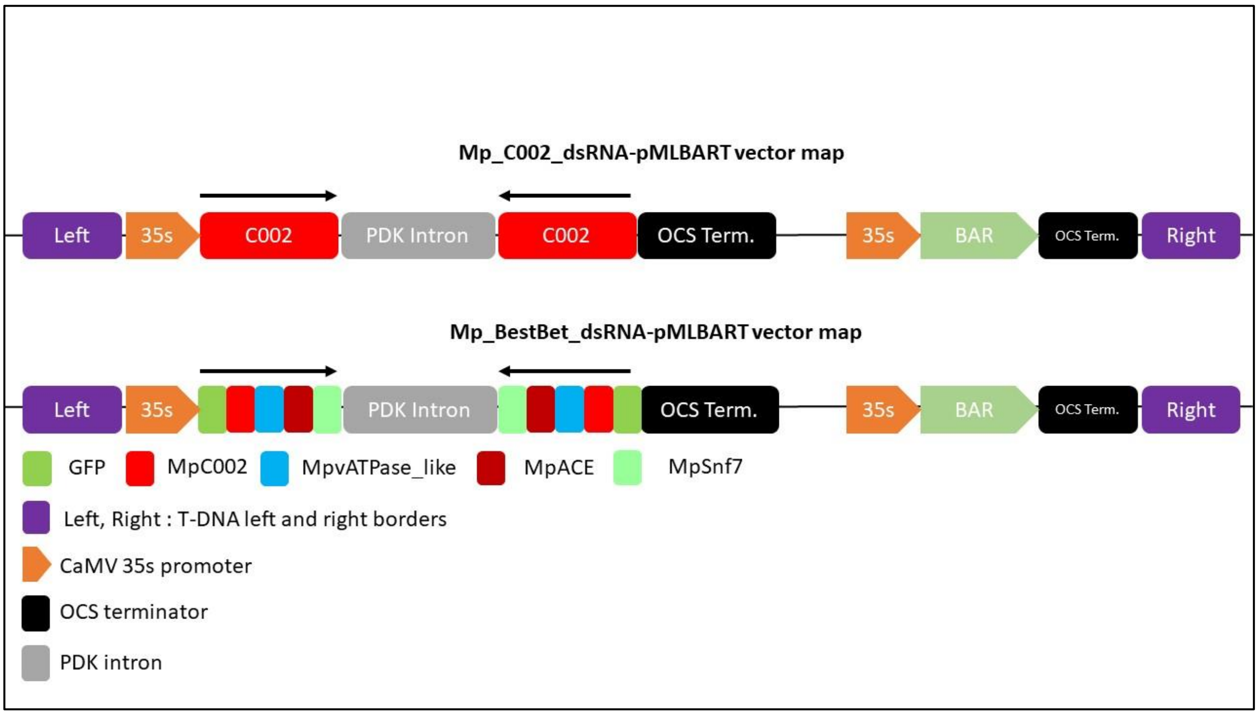

Supplementary Figure 3 – Plant transformation vector maps. The vector on the left is designed to express dsMp*C002 and* the vector on the right is designed to express the multigenic dsBestBet (*C002, vATPAse-like, Ace, snf7*).

Supplementary Figure 4: Phylogeny of Ferritin heavy chain

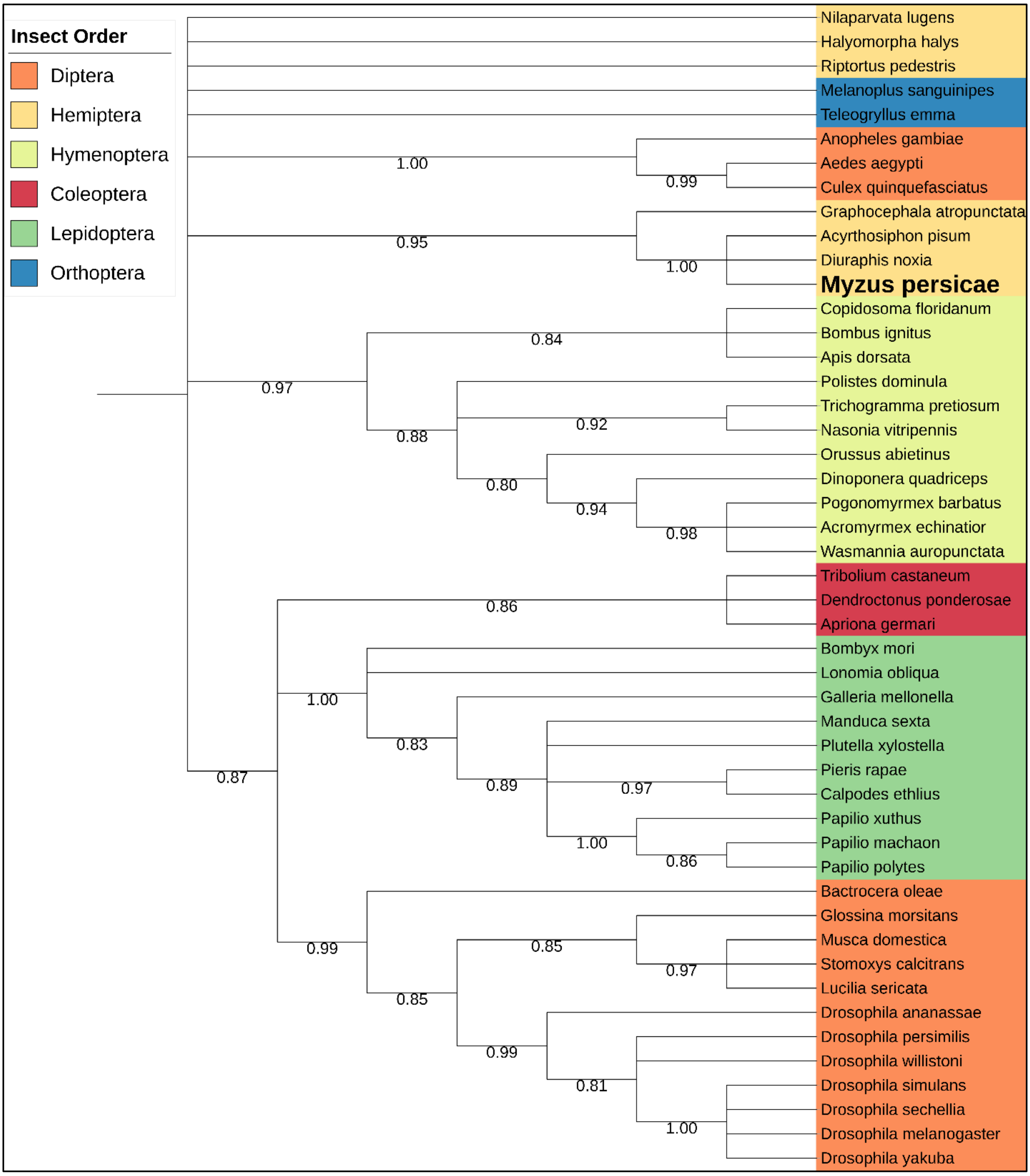

Supplementary figure 4: The phylogeny of ferritin heavy chain genes.

Supplementary Figure 5

Effect of Iron on aphid size –

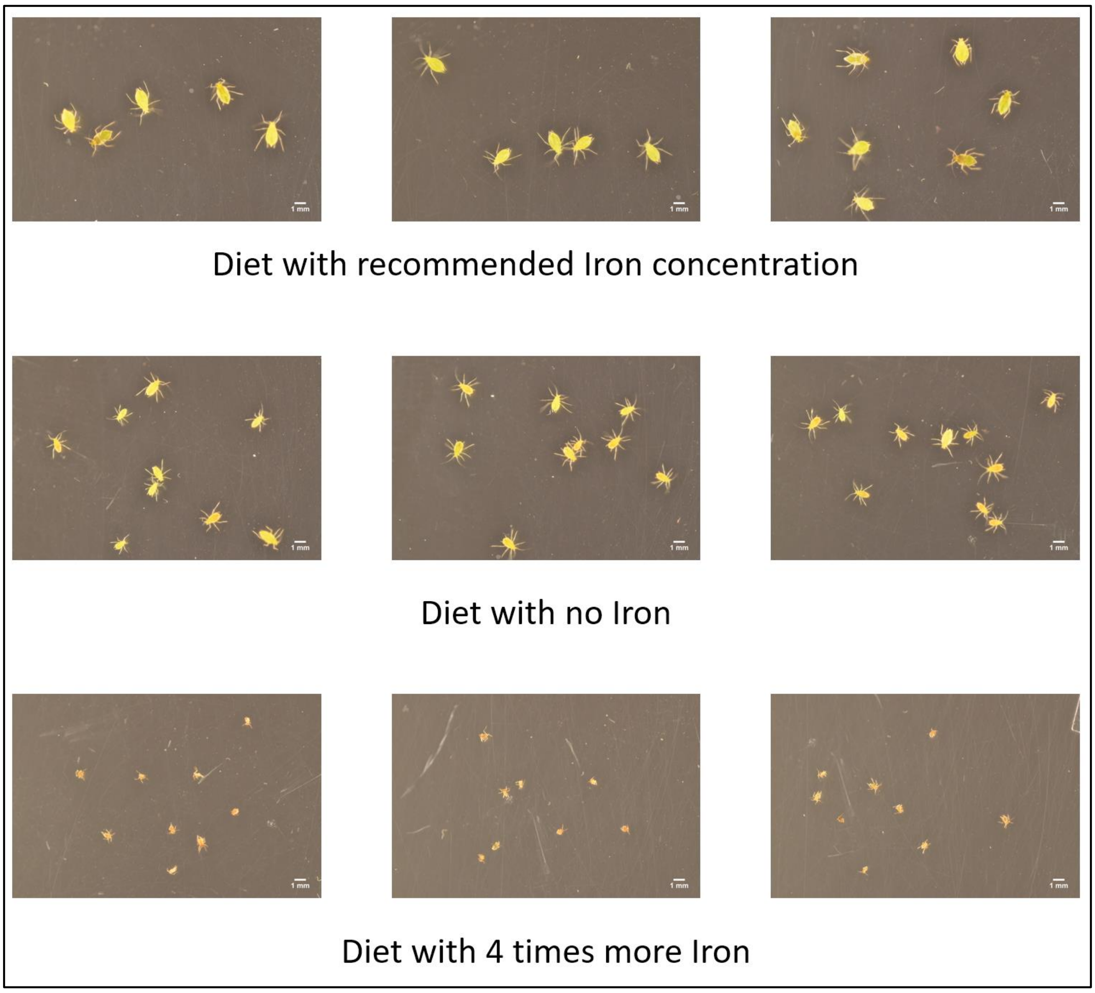

Supplementary figure 5: Photographs showing effect of different concentrations of iron on *Myzus persicae* size.

Supplementary Figure 6: Primers for C002 analysis

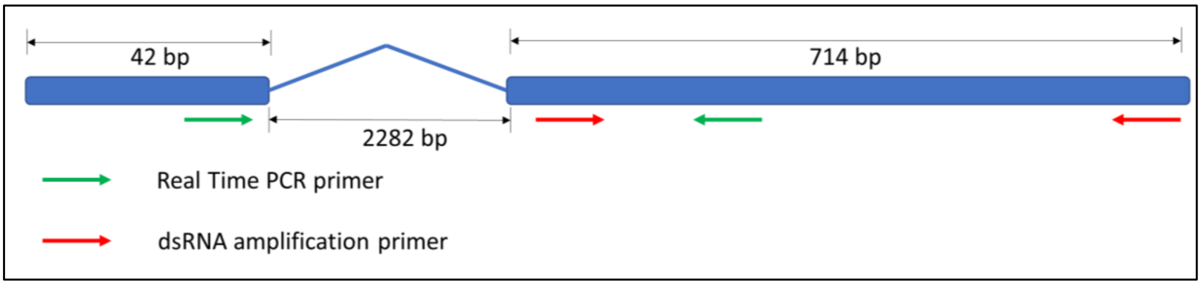

Supplementary figure 6: Physical location *dsMP_C002* primer used for amplification of dsRNA and qRT-PCR analysis.

Supplementary Figure 7: Phylogeny of Endonucleases of insects

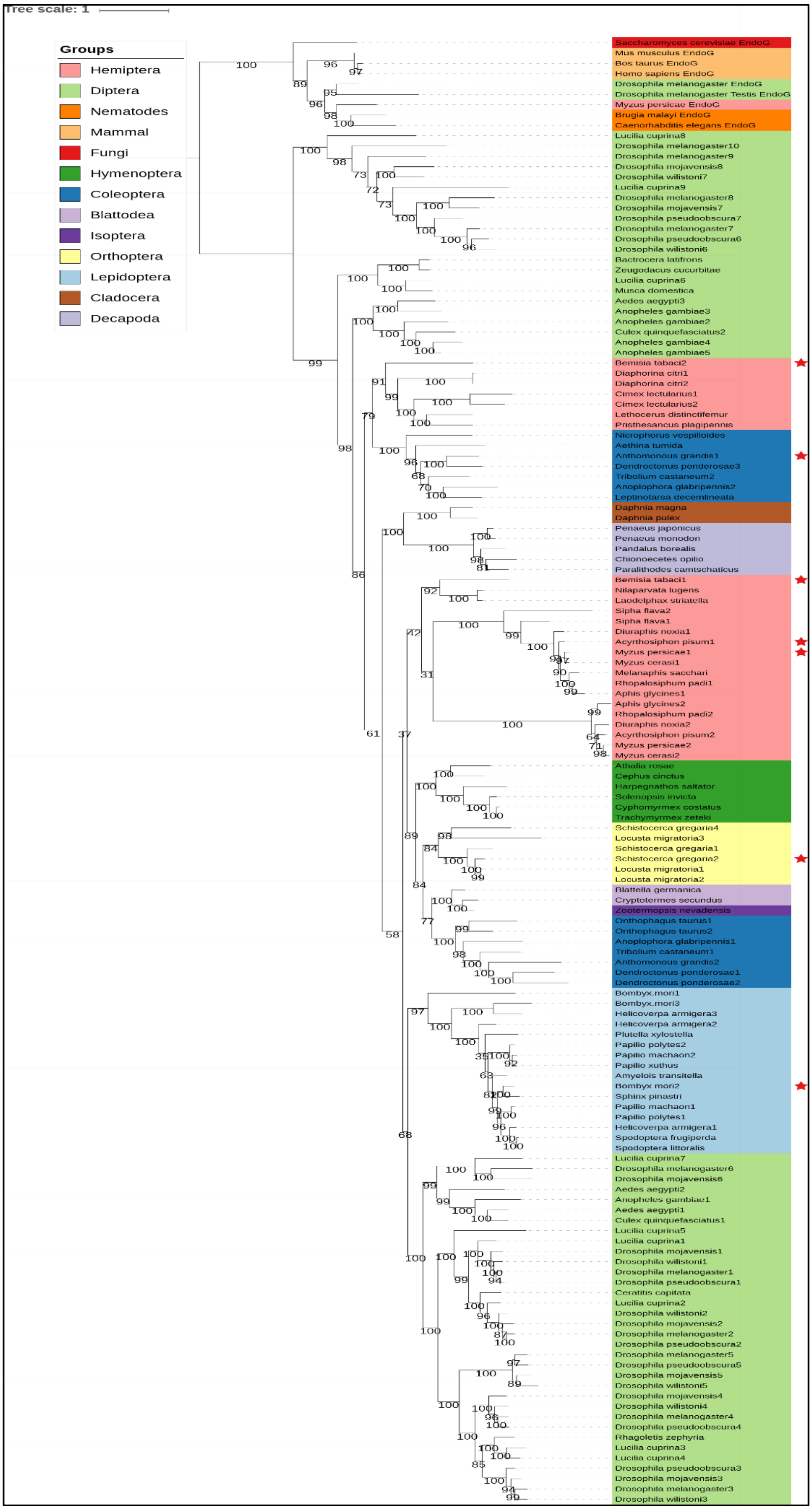

Supplementary figure 7: An extended maximum likelihood phylogenetic tree of a subset of insect extracellular endonucleases. Bootstrap scores are shown as percentages. The asterisks represent sequences that have reported dsRNA activity in the literature. The endo-G clade was specified as an outgroup (all accession numbers in supplementary excel sheet1).

Supplementary Figure 8: Time course of dsRNA degradation with/without citrate

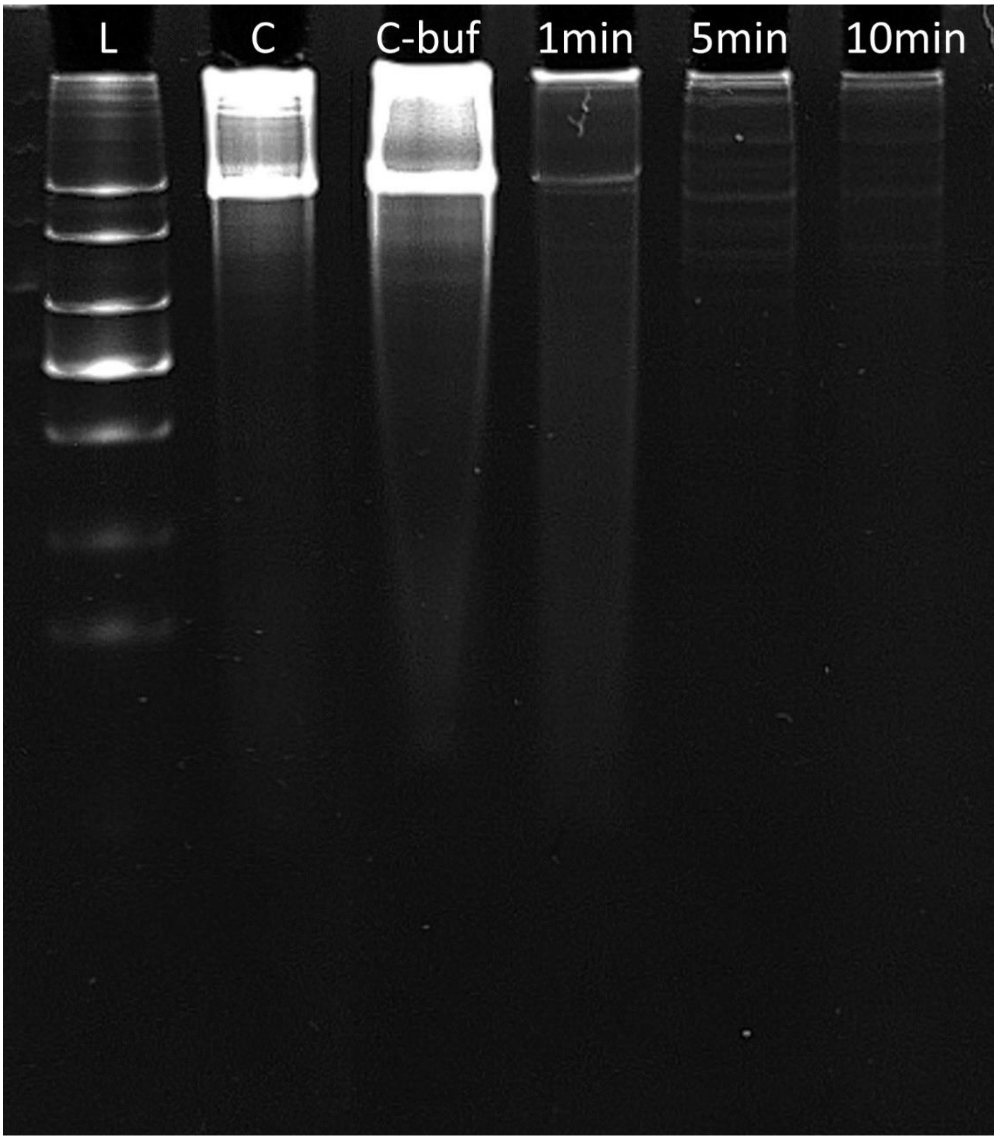

Supplementary Figure 8: Inhibition of dsRNA degradation by standard saline citrate that inhibits the action of nuclease. C = Control dsRNA at 0 min, C-buf = dsRNA with neutral saline citrate incubated with *M. persicae* midgut for 15 min, 1min = dsRNA incubated with *M. persicae* midgut for 1 minute, 5min = dsRNA incubated with *M. persicae* midgut for 5 minutes, dsRNA incubated with *M. persicae* midgut for 10 minutes. L = ladder 50bp.

Supplementary Table 1:

Gene Name	Top blast hit from AphidBase transcript data	Similar Cluster	RPKM and Std Err in MG	RPKM and Std Err in WB
MpACE	MYZPE13164_G006_v1.0_000103290.1	Cluster-5961.1	1.8±0.1	2.7±0.1
MpC002	MYZPE13164_G006_v1.0_000086200.2	Cluster-32898.1	2±0.2	154.4±8.9
MpSnf7	MYZPE13164_G006_v1.0_000183180.2	Cluster-23766.0	32.7±0.5	16.2±0.3
MpvATPase_like	MYZPE13164_G006_v1.0_000172560.3	Cluster-28471.1	9.8±0.2	5.4±0.1
MpvATPase	MYZPE13164_G006_v1.0_000095950.1	Cluster-21274.0	944±39.6	178.7±2.6
MpFerritin	MYZPE13164_G006_v1.0_000072220.2	Cluster-26442.0	1842±285.5	255.5±4.7
MpGADPH	MYZPE13164_G006_v1.0_000000720.1	Cluster-35712.0	2413.6±71	1161.8±60.4
MpTubulin	MYZPE13164_G006_v1.0_000052180.2	Cluster-37187.0	345.8±12.5	821.7±29.7
MpRpL7	MYZPE13164_G006_v1.0_000098680.1	Cluster-27421.0	917.5±38.1	635.2±13.4

Supplementary Table 1: The transcript abundance of genes studied in this manuscript. (MigGut: MB, Whole Body: WB, RPKM: Reads Per Kilobase of transcript per Million mapped reads, Std Err: Standard Error, FC: Fold Change).

Supplementary data 1: All the dsRNA sequences and primers used in the study.

Artificially synthesized dsRNA sequences

>*Mp_C002_dsRNA*

5’TGAACGATAATCAGGGAGAAGAGAACGATAATCAGGGAGAAGAGAACGATAATCAGGGAGAAGAGAACG
ATAATCAGGGAGAAGAGAAGGAAGAAGTTTCCGAACCAGAGATGGAGCACCATCAGTGCGAAGAATACAAA
TCGAAGATCTGGAACGATGCATTTAGCAACCCGAAGGCTATGAACCTGATGAAACTGACGTTTAATACAGCTA
AGGAATTGGGCTCCAACGAAGTGTGCTCGGACACGACCCGGGCCTTATTTAACTTCGTCGATGTGATGGCCAC
CAGCCCGTACGCCCACTTCTCGCTAGGTATGTTTAACAAGATGGTGGCGTTTATTTTGAGGGAGGTGGACACG
ACATCGGACAAATTTAAAGAGACGAAGCAGGTGGTCGACCGTATCTCGAAAACTCCAGAGATCCGTGACTAT
ATCAGGAACTCGGCCGCCAAGACCGTCGACTTGCTCAAGGAACCCAAGATTAGAGCACGACTGTT 3’

>*Mp_FeHC_dsRNA*

5’GGTCCAAATTCCAGACGATTGGATCACCATGGTTGACCCATGCACGAAAAAAATGAAAGAGCAGGTCCAGG
AAGAACTTACTGCAGCAATGACATATTTTGCAATGGGAGCACATTTTTCTAAAGACACAGTGAATCGTCCAGG
ATTCGCAAAAATCTTCTTTGATAGTGCCAGCGAAGAACGTGACCATGCTATTAAAATTATTGGATATCTGTTGA
TGAGAGGAGGTTTGACCAAAGATATCAGTCAATTAATTCGTGACCCTCAACCTTTGTCTGAAGCATGGGCTGA
TGGTCTAAGTGCATTGAAAGATGCTTTAAAATTGGAAGCTCATGTCACTCGTAAGATAAGAGATATTGCCACC
ACATGTGAGGAACCTGGACGTGATGGACAAGATTTCAACGACTATCATTTAGTTGATTGGTTAACTGGCGATT
TCTTGACTGAACAATATGAAGGTCAGCGTGACTTAGCT 3’

>*Dm_vATPase_dsRNA*

5’AAGGCCCAGATCAATCAGAACGTCGAGCTGTTCATCGACGAGAAAGACTTCCTCTCTGCTGATACCTGCGGT
GGTGTTGAGCTGCTGGCCCTCAACGGACGCATCAAGGTGCCCAATACGCTGGAGTCCAGATTAGACCTCATTT
CGCAGCAGCTGGTGCCCGAGATTCGTAACGCACTTTTCGG 3’

>*Mp_C002_dsRNA* expressed in Arabidopsis

5’-
GGGGTACCTGCAAGGTTCAGACTTCCGAACAGGACGATGATCAGGAAGGATATTACGATGATGAGGGAGGA
GTGAACGATAATCAGGGAGAAGAGAACGATAATCAGGGAGAAGAGAACGATAATCAGGGAGAAGAGAACG
ATAATCAGGGAGAAGAGAAGGAAGAAGTTTCCGAACCAGAGATGGAGCACCATCAGTGCGAAGAATACAAA
TCGAAGATCTGGAACGATGCATTTAGCAACCCGAAGGCTATGAACCTGATGAAACTGACGTTTAATACAGCTA
AGGAATTGGGCTCCAACGAAGTGTGCTCGGACACGACCCGGGCCTTATTTAACTTCGTCGATGTGATGGCCAC
CAGCCCGTACGCCCACTTCTCGCTAGGTATGTTTAACAAGATGGTGGCGTTTATTTTGAGGGAGGTGGACACG
ACATCGGACAAATTTAAAGAGACGAAGCAGGTGGTCGACCGTATCTCGAAAACTCCAGAGATCCGTGACTAT
ATCAGGAACTCGGCCGCCAAGACCGTCGACTTGCTCAAGGAACCCAAGATTAGAGCACGACTGTTCAGAGTG
ATGAAAGCCTTCGAGAGTCTGATAAAACCAAACGAAAACGAAGCATTAATCAAACAGAAGATTAAGGGGTTA
ACCAATGCTCCCGTCAAGTTAGCCAAGGGTGCCATGAAAACGGTTGGACGTTTCTTTAGACATTTTTAACGGA
ATTC-3’

>*Mp_Bestbet_dsRNA* expressed in Arabidopsis

5’-
ACGACGGGAACTACAAGACACGTGCTGAAGTCAAGTTTGAGGGAGACACCCTCGTCAACAGGATCGAGCTTA

AGGGAATCGATTTCAAGGAGGACGGAAACATCCTCTAGACTCGAGAGGAATTGGGCTCCAACGAAAGTGTGC
TCGGACACGACCCGGGCCTTATTTAACTTCGTCGATGTGATGGCCACCAGCCCGTACGCCCACTTCTCGCTAGG
TATGTTTAACAAGATGGTGGCGTTTATTTTGAGACGGGCAAAGGTCGAAGGTCAATGGTTGAAAATACGCTG
GTAGTGCGATTGCTCCATCTCACCCAACAAGCTATACCGTTGATATGCACTGGACTGTTTGGACTGAATCCCAC
TAGAACTTACTCAAGACATAGATAAGATAGCAACCCGATTGCCACCATGTCGTGCATGAGAGCTGTTGACGCA
AGTACCATATCCAAAAAGCAGTGGAACAGCTATTCCGGTATTTTGGGTTTCCCGTCTGCGCCCACCGTGGAGG
CGTTCTCCTTCCGGAACACCCGCTGGACATGTTGAAATGGTACAACTAATAAACGAGCTGCATTGCAAGCATT
GAAGCGTAAGAAACGGTACGAACAACAATTAGCCCAAATTGATGGTTCCATGTTAACTATTGAACAACAGCG
GGAGGCATTAGAAGGTGCCAACACAA-3’

>*Mp_vATPase_like_dsRNA in vitro* transcribed

5’-
TTTAGCCAACACGGGAATGAACATCAAAATAAACATTGACCAAGCAACTAAGTTACCGACTCAAGAAATAGGA
GGCGTCGTGGTCACGGGCAAAGGTCGAAGGTCAATGGTTGAAAATACGCTGGTAGTGCGATTGCTCCATCTC
ACCCAACAAGCTATACCGTTGATATGCACTGGACTGTTTG-3’

Supplementary data 2: All Primers used in this Study

**Aphid qPCR primers**	**Forward**	**Reverse**
qMpGAPDH	5’GTCTTCCGACTTCATTGGTGATA3’	5’CCACAACACGGTTGGAGTAA3’
qMpRpL7	5’GGACAACGCATTCCAATCAC3’	5’CTTGAATCGACGATCAGCTCTA3’
qMpRpLS3	5’CCATGGGACCAAACAGGTAAA3’	5’GGAACTGCTACTTCGGGTTTAG 3’
qMpTubulin	5’CGGTTTCCCGGTCAGTTAAA3’	5’CTAGCTGTAAGTGGAGCGAATC 3’
qMpActin	5’CCGAAAGAGGTTACAGCTTCA3’	5’TCGAAGTCCAAAGCGACATAG3’
qMpvATPase_like	5’CTACAGGCGATGTATCAAATCTT3’	5’TGATGTTCATTCCCGTGTTG3’
qMpC002	5’GCTCAAGGAACCCAAGATTAGA3’	5’TAACTTGACGGGAGCATTGG3’
qMpFerritin	5’CTGGACGTGATGGACAAGATT3’	5’AAGTCACGCTGACCTTCATATT3’
**Arabidopsis primers for internal control**		
qCyclophilin_AT	5’CACTGGACCAGGTGTACTTTC3’	5’ACCACATGCCTTCCATCTAAC3’
qTCTP_AT	5’ACACCCAAGCTCAGCGAAGAA3’	5’CATGCATACCCTCCCCAACAA3’
qTubulin_AT	5’CATTTGCTTCGGTACACTCCA3’	5’ CCAGGGAACCTAAGACAGCA3’
qActin_AT	5’TCTTCCGCTCTTTCTTTCCA3’	5’TCCTTCTGGTTCATCCCAAC3’
**Digital PCR Primers and probes**		
MpRpL7_digi	5'AATGAACTTCCTTTGGCCTTTC3'	5'GTTAATGGTATCTTCACGGTTTC C3'
MpRpL7_digi_Probe	5'GATTGGTCTTCTTACGCCAGCCTCC3'	
MpC002_digi	5'CCATCAGTGCGAAGAATACAAATC3'	5'GTTGGAGCCCAATTCCTTAGC3'
MpC002_digi_Probe	5'TCAGGTTCATAGCCTTCGGGTTGC3'	
MpvATPase_like_digi	5'CTACAGGCGATGTATCAAATCTT3'	5'TGATGTTCATTCCCGTGTTG3'
MpvATPase_like_probe	5'TACCAGACGACGTGGAGTACGTGA3'	
MpACE_digi	5'ATGGAAGCGTTCACCAGATT3'	5'TCCGTAACCTACGTGCAATTC3'
MpACE_digi_prob	5'CTGTCTCAGATGACATAGATTGGCCA CT3'	
MpSnf7_digi	5'GGAACTGATGTAGACGAGGATG3'	5'GCTCAAATGTTGGAACTGAAGG 3'
MpSnf7_digi_prob	5'AGGCAAGACACCTGTCCA3'	

Supplementary data 3: Custom commands used in bioinformatics analysis pipeline.

Custom Bioinformatics Commands –

Trimmomatic

```
java -jar trimmomatic-0.33.jar PE -threads 14 <fastq_file_R1>
<fastq_file_R2> fastq_paired_R1.fastq fastq_unpaired_R1.fastq
fastq_paired_R2.fastq fastq_unpaired_R2.fastq ILLUMINACLIP:TruSeq3-
PE-2.fa:2:30:10 LEADING:3 TRAILING:3 MINLEN:40
```

STAR –

```
STAR --runThreadN 16 --runMode alignReads --sjdbGTFfile <GTF_file> -
-sjdbOverhang 99 --outSAMstrandField intronMotif --twopassMode Basic
--quantMode GeneCounts --readFilesIn <Read_1.fastq> <Read_2.fastq> -
-outFilterMatchNminOverLread 0.4 --outFilterScoreMinOverLread 0.2 --
outReadsUnmapped Fastx --limitBAMsortRAM 8729257684 --genomeDir . --
outSAMtype BAM SortedByCoordinate --outSAMunmapped Within --
outFilterIntronMotifs RemoveNoncanonical > star.log
```

Supplementary data 4: Link for aphid size detection script at GitHub.

Link - https://github.com/amoltej/Aphid_Ruler

Supplementary data 5: Sequence accession numbers used in phylogenetic study

Name of sequence	Accession number
Myzus persicae EndoG	XP_022164839.1
Drosophila melanogaster EndoG	NP_610737.1
Caenorhabditis elegans EndoG	T28787
Brugia malayi EndoG	XP_001892352.1
Homo sapiens EndoG	AAP88743.1
Bos taurus EndoG	NP_787017.1
Mus musculus EndoG	NP_031957.1
Drosophila melanogaster Testis EndoG	ACN22207.1
Saccharomyces cerevisiae EndoG	NP_012327.1
Aedes aegypti1	EAT42072.1
Culex quinquefasciatus1	XP_001858177.1
Anopheles gambiae1	XP_309696.2
Aedes aegypti2	XP_001648469.1
Drosophila wilistoni1	XP_002068162.1
Drosophila melanogaster1	NP_649078.1
Drosophila pseudoobscura1	XP_001352914.1
Drosophila mojavensis1	XP_002008430.1
Lucilia cuprina1	KNC28514.1
Drosophila wilistoni2	XP_002068164.1
Drosophila melanogaster2	NP_649076.1
Drosophila pseudoobscura2	XP_001352912.1
Drosophila mojavensis2	XP_002008429.1
Lucilia cuprina2	XP_023303709.1
Ceratitis capitata	XP_004530585.1
Drosophila wilistoni3	XP_002062101.1
Drosophila mojavensis3	XP_002008700.1
Drosophila pseudoobscura3	XP_002021311.1
Drosophila melanogaster3	NP_648610.1
Drosophila wilistoni4	GK16832c
Drosophila pseudoobscura4	XP_001353493.2
Drosophila melanogaster4	ABC86341.1
Drosophila mojavensis4	GI13641c
Rhagoletis zephyria	XP_017478492.1
Lucilia cuprina3	XP_023306795.1
Lucilia cuprina4	XM_023451027.1
Drosophila wilistoni5	GK16832b
Drosophila mojavensis5	GI13641b
Drosophila pseudoobscura5	GA12772b
Drosophila melanogaster5	CG14120b
Lucilia cuprina5	XP_023303878.1
Athalia rosae	XP_012258516.1
Cephus cinctus	XP_015605998.1
Onthophagus taurus1	XP_022903813
Onthophagus taurus2	XP_022903812
Blattella germanica	PSN57821.1
Zootermopsis nevadensis	KDR18788.1
Cryptotermes secundus	XP_023717429.1
Schistocerca gregaria1	KJ135008
*Schistocerca gregaria2	KJ135009
Locusta migratoria1	APF31794
Locusta migratoria2	ARW74135

Trachymyrmex zeteki	XP_018306713.1
Cyphomyrmex costatus	KYM98744.1
Solenopsis invicta	XP_011156845.1
Harpegnathos saltator	EFN78781.1
Tribolium castaneum1	D6WCE2
Anoplophora glabripennis1	XM_018724288.1
Nilaparvata lugens	XP_022200679
Laodelphax striatella	AKB95592
*Bemisia tabaci1	KX390872
Drosophila ponderosae1	N6UQ21
Drosophila ponderosae2	N6UIT3
Anthomonous grandis2	Agranuc1
Schistocerca gregaria4	KJ135011
Sphinx pinastri	Sphinx_pinastriC433742
*Bombyx mori2	AB254196
Papilio machaon1	XM_014500085.1
Papilio polytes1	XM_013279368.1
Spodoptera frugiperda	CAR92521.1
Spodoptera littoralis	CAR92522.1
Helicoverpa armigera1	XM_021336132
Amyelois transitella	XM_013330480.1
Papilio machaon2	XP_014355571.1
Papilio xuthus	XM_013315183.1
Papilio polytes2	XM_013283730.1
Helicoverpa armigera2	XM_021336155
Plutella xylostella	XM_011571062.1
Helicoverpa armigera3	XM_021335286
Bombyx.mori3	XP_004922835
Daphnia pulex	EFX68769.1
Daphnia magna	KZS08845.1
Bombyx.mori1	AK383943.1
Chionoecetes opilio	BAI79321.2
Paralithodes camtschaticus	AAN86143.1
Penaeus japonicus	CAB55635.1
Penaeus monodon	DQ528804.2
Pandalus borealis	CBG22733.1
Aedes aegypti3	XP_021699422.1
Anopheles gambiae3	XP_315045.4
Anopheles gambiae4	XP_320812.4
Anopheles gambiae5	XP_320813
Culex quinquefasciatus2	XP_001859639.1
Anopheles gambiae2	XP_320813.4
Tribolium castaneum2	EEZ98722
Leptinotarsa decemlineata	XM_023168151
Anoplophora glabripennis2	XM_023455638
Drosophila ponderosae3	XM_019907742
*Anthomonous grandis1	Agranuc2
Aethina tumida	XM_020014593
Nicrophorus vespilloides	XM_017918580
Locusta migratoria3	ARW74134
Pristhesancus plagipennis	AQM58352
Lethocerus distinctifemur	ATU82404

Cimex lectularius1	XP_014241376
Cimex lectularius2	XP_014241898
Diaphorina citri1	XP_008483860
Diaphorina citri2	XP_008483858
*Bemisia tabaci2	AQU43107
Musca domestica	XP_005182498.1
Lucilia cuprina6	XP_023293712.1
Bactrocera latifrons	XP_018802082.1
Zeugodacus cucurbitae	XP_011187184.1
Drosophila mojavensis6	XP_002008704.2
Drosophila melanogaster6	NP_648612.1
Lucilia cuprina7	FF38_05889
Diaphorina citri3	XP_017297751.1
Diuraphis noxia1	XP_015375167.1
**Myzus persicae1	XP_022183034.1
Myzus cerasi1	Mca21568.t1
*Acyrthosiphon pisum1	XP_003242652.1
Rhopalosiphum padi1	Rp154-scaffold
Melanaphis sacchari	XP_025194442.1
Aphis glycines1	AG012784
Sipha flava1	XP_025416859.1
Sipha flava2	XP_025421666.1
Myzus persicae2	XP_022181213.1
Diuraphis noxia2	XM_015519681.1
Acyrthosiphon pisum2	XP_003248225.1
Aphis glycines2	AG007731
Rhopalosiphum padi2	Rp463-scaffold
Myzus cerasi2	Mca00011.t1
Drosophila pseudoobscura6	XP_001357871.2
Drosophila melanogaster7	NP_001034074.1
Drosophila wilistoni6	XP_002070514.1
Drosophila pseudoobscura7	XP_002137045.2
Drosophila mojavensis7	XP_002004969.1
Drosophila melanogaster8	NP_610558.2
Drosophila wilistoni7	EDW81498.2
Drosophila mojavensis8	XP_002001066.2
Drosophila melanogaster9	NP_996301.2
Drosophila melanogaster10	NP_651627.1
Lucilia cuprina8	XP_023306757.1
Lucilia cuprina9	XP_023292190.1
